# Reactive oxygen species/hypoxia dual-responsive polymers combined with melatonin inhibited PANoptosis of retinal ganglion cells for acute glaucoma treatment

**DOI:** 10.7150/thno.112836

**Published:** 2026-01-01

**Authors:** Shuhan Meng, Weizhou Fang, Yukun Wu, Zhaohua Xia, Tiansheng Chou, Yang Xia, Lexi Ding, Xuezhi Zhou, Xiaobo Xia

**Affiliations:** 1 Eye Center of Xiangya Hospital, Central South University, Changsha, Hunan, 410008, P. R. China.; 2 Hunan Key Laboratory of Ophthalmology, Changsha, Hunan, 410008, P. R. China.; 3 National Clinical Key Specialty of Ophthalmology, Changsha, Hunan, 410008, P. R. China.; 4 National Medical Metabolomics International Collaborative Research Center, Xiangya Hospital, Central South University, Changsha, Hunan, 410008, P. R. China.; 5 National Clinical Research Center for Geriatric Disorders, Xiangya Hospital, Central South University, Changsha, Hunan, 410008, P. R. China.

**Keywords:** glaucoma, retinal ganglion cell, PANoptosis, melatonin, nanoparticle

## Abstract

**Rationale:** Acute glaucoma is triggered by sudden spikes in intraocular pressure, which induces retinal ischemia/reperfusion (RI/R), leading to hypoxia, oxidative stress, and ultimately PANoptosis in retinal ganglion cells (RGCs). Developing a therapeutic approach that simultaneously targets these events may offer a promising strategy for reducing secondary neuronal damage in acute glaucoma.

**Methods:** We developed a reactive oxygen species (ROS)/hypoxia dual-responsive, biodegradable nanoparticle system (NPs) containing azo and thioketal bonds, designed to encapsulate melatonin (MT), a known endogenous antioxidant and PANoptosis inhibitor. The biocompatibility, biosafety, and therapeutic efficacy of MT-NPs were evaluated *in vitro* using an oxygen-glucose deprivation/reperfusion (OGD/R) R28 cell model and *in vivo* using a RI/R rat model.

**Results:** The NPs efficiently released encapsulated MT in response to hypoxic conditions and the presence of ROS. This controlled-release system improved both the biocompatibility and long-term retention of MT in the retina. MT-NPs effectively alleviated hypoxia, cleared excess ROS, and inhibited PANoptosis in RGCs following acute glaucomatous injury. Compared to direct MT administration, MT-NPs were more effective at protecting RGC axons and somas and facilitating restoration of visual function in rats with acute glaucoma.

**Conclusion:** This simplified but multifunctional delivery system leveraged the widely available and safe compound melatonin in a highly efficient nanoparticle platform. This system offers potent neuroprotective effects to the retina preventing injury caused by acute glaucoma, and thereby providing a promising clinically translatable strategy for the treatment of glaucoma.

## Introduction

Glaucoma is an irreversible, neurodegenerative disease characterized by the progressive death of retinal ganglion cells (RGCs) and degeneration of axons 1. This condition, which is primarily caused by pathologically high intraocular pressure (IOP), is expected to affect more than 100 million individuals by 2040 1, 2. Acute primary angle-closure glaucoma (APACG), triggered by a sudden and substantial increase in IOP, remains a common type of glaucoma among Asians and is estimated to affect 20 million people worldwide 3, 4. The rapid increase in IOP induces retinal ischemia/reperfusion (RI/R) injury, eventually triggering an event cascade that exacerbates RGC damage 5. Currently, glaucoma treatment predominantly involves pharmacological treatments, laser procedures, and surgical interventions aimed at reducing IOP 6. However, despite effective IOP reduction, numerous patients continue to experience disease progression 7. This is because IOP control alone cannot mitigate the persistent degenerative process affecting RGCs, which potentially involves oxidative damage and cell death pathway activation 8. Therefore, developing effective neuroprotective strategies against glaucoma is imperative.

PANoptosis is a newly identified type of programmed death characterized by simultaneous activation of apoptosis, necroptosis, and pyroptosis 9. In animal models of glaucoma, the pathways involved in RGC death include BAX/Caspase-dependent apoptosis 10-12, receptor interacting protein kinase 1/3 (RIPK1/3)/ mixed lineage kinase-like domain protein (MLKL)-dependent necroptosis 13, 14, and NOD-like receptor pyrin domain-containing protein 3 (NLRP3)/Caspase-1/Gasdermin D (GSDMD)-dependent pyroptosis 15, 16. Therefore, PANoptosis might play a central role in RGC death during glaucomatous neurodegeneration.

Reactive oxygen species (ROS) accumulation and PANoptosis activation may be closely associated processes 17. Excessive ROS generation can be induced by a hypoxic environment typically occurring following ischemia/reperfusion injury 18. Previous research indicates that excessive ROS accumulation promotes apoptosis by inducing mitochondrial translocation and BAX activation 19, 20. ROS accumulation also induces RIPK1 phosphorylation and activation, triggering necroptosis 21. Moreover, ROS activates NLRP3, which contributes to pyroptosis 22-24. However, recent studies have reported that genetic or pharmacological inhibition of apoptosis, necroptosis, and pyroptosis pathways decreases ROS production 25-27. Thus, a positive feedback loop likely exists between ROS production and PANoptosis induction. Furthermore, ROS directly cause oxidative damage to DNA, proteins, and lipids 28. Therefore, removal of ROS and inhibition of cellular PANoptosis may effectively reduce RGC death in patients with glaucoma.

Recent research in glaucoma therapy have primarily explored two categories of experimental neuroprotection approaches. The first category involves using antioxidants to scavenge ROS in RGCs 29, 30. However, simple molecule-based antioxidants are limited by their low transportation potency and short duration of action. In contrast, sophisticated ROS-scavenging biomaterials and nanomaterials, such as photosensitizer-containing polymers, may produce various side effects and are difficult to synthesize 31-33. The second category involves using gene therapy or small-molecule drugs to selectively inhibit the activation of cell death pathways 34-36. Nevertheless, these strategies are limited by off-target effects, slow onset, and specificity of single-death pathways, reducing their practical neuroprotective efficacy in RGCs 37, 38. The clinical application of many current treatment strategies is limited by major toxic side effects and the induction of complex metabolic pathways that produce deleterious metabolites. An innovative and simple design of a drug delivery system combined with effective drugs already used clinically may be an ideal strategy for glaucoma treatment.

Melatonin (MT; N-acetyl-5-methoxytryptamine, C_13_N_2_H_16_O_2_) is an indole neuroendocrine hormone primarily secreted by the pineal gland 39, and is recognized for its role in treating insomnia through interactions with the suprachiasmatic nucleus of the hypothalamus and the retina 40. MT has several beneficial attributes including antioxidant and anti-inflammatory properties 41. Moreover, exogenous MT supplementation has demonstrated substantial neuroprotective effects in the treatment of various neurodegenerative diseases including Alzheimer's disease 42-44, Parkinson's disease 45, 46, traumatic brain injury 47, and stroke 48. Furthermore, accumulating evidence supports MT's ability to inhibit apoptosis 49, 50, necroptosis 51, and pyroptosis 52. In hypoxia-induced retinopathy, exogenous MT administration protects RGCs from apoptosis and improves visual function 53, 54. We have previously shown that exogenous MT supplementation protects RGCs against glutamate-induced retinal excitotoxicity 55. These findings strongly suggested that MT potentially may perform neuroprotective functions in glaucoma. Crucially, MT has a good safety profile and has been approved as a dietary supplement in several countries. However, the therapeutic efficacy of MT is limited by its short half-life (<30 min), making dosing complex, and it is further compounded by issues related to administration 56. Thus, the development of a novel delivery system for localized and controlled MT delivery is essential.

In this study, a nanoparticle (NP) made of a biodegradable polymer featuring azo and thioketal bonds in its backbone structure was developed. These bonds facilitate disassembly and cargo release in response to hypoxic and ROS-rich conditions. Specifically, breakage of the azo bonds alleviates hypoxia, and disruption of the thioketal bonds consumes ROS. We encapsulated the PANoptosis inhibitor MT within NPs to create MT-NPs. To evaluate the biological effects of MT-NPs, we constructed an oxygen-glucose deprivation/reperfusion (OGD/R) R28 cell model (R28^OGD/R^) and a RI/R rat model (Rat^RI/R^) to mimic acute glaucomatous injury *in vitro* and *in vivo*, respectively. In our study, MT-NPs demonstrated high biocompatibility, long-term retention, and promising neuroprotective effects. Compared to free MT, MT-NPs were more effective in alleviating hypoxia, scavenging excessive ROS, and inhibiting PANoptosis activation in RGCs, resulting in superior RGC axon and soma protection and improved visual function after RI/R injury. Overall, this study provides a novel, simplified MT drug delivery system that offers excellent RGC protection in response to acute glaucomatous injury. Therefore, our MT drug delivery system may be considered a reliable strategy for clinical glaucoma treatment.

## Materials and Methods

### Materials and equipment

3,3'-(Propane-2,2-diylbis(sulfanediyl))dipropionic acid (PDSD), (E)-(diazene-1,2-diylbis(4,1-phenylene))dimethanol (DDPD), and N, N'-carbonyldiimidazole (CDI) were purchased from Bide Pharmatech. mPEG5000OH was purchased from Aladdin. Dulbecco's modified Eagle's medium (DMEM) (#PM150220), Trypsin (#25300054) and serum were purchased from Gibco. LDH release assay kit (#C0019S), CCK8 (#C0037), 2',7'-dichlorodihydrofluorescein (DCFH-DA, #S0033S), Calcein AM/PI double staining kit (#C2015M) and PI/Annexin V-FITC Apoptosis detection kit (#C1062M) were purchased from Beyotime. Hypoxyprobe was purchased from Hypoxyprobe, Inc. (Burlington, USA). BAX (#60267-1-Ig), cleaved-Caspase 3 (#19677-1-AP, #25128-1-AP), cleaved-Caspase 7(#27155-1-AP), RIPK1 (#17519-1-AP), GSDME N-Terminal (N-GSDME, #13075-1-AP) and β-actin (66009-1-Ig) antibody were purchased from Proteintech. RIP3 (#95702) were purchased from Cell Signaling Technology. p-RIP3 (#AF7443**),** NLRP3 (#DF7438) and GSDMD N-Terminal (N-GSDMD, #DF13758) antibody were acquired from Affinity Biosciences. p-MLKL antibody (#44213) was purchased from Signalway Antibody. RBPMS antibody was purchased from GeneTex (#118619). 4′,6-diamidino-2-phenylindole (DAPI, #C1005) was acquired from Beyotime. RIPA buffer (#R0010) and protein loading buffer (#P1040) were purchased from Solarbio. A cocktail of protein phosphatase and protease inhibitors were purchased from Sigma Aldrich. SurePAGE Gels were purchased from GenScript. PVDF membrane was from MILLIPORE. IL-1β ELISA kit was purchased from Proteintech (#KE20021). Terminal deoxynucleotidyl transferase dUTP nick end labeling (TUNEL) assay kit (#C1086), LDH release assay kit (#C0019S), Caspase-1 activity assay kit (#C1101) and Caspase-3 activity assay kit (#C1115) were purchased from Beyotime. Flow cytometric analysis was performed using a flow cytometer from BD Biosciences. The confocal microscope system (Zeiss LSM 900) was produced by Carl Zeiss. The H&E imaging analysis was performed using an inverted microscope (Olympus IX 83). Tanon 5200 Multi fully automated chemiluminescence image analysis system was used for the western blotting analysis. Quantitative determination of CCK8 was carried out using a Bio-Rad microplate reader. LC-MS instruments were obtained from Thermo Fisher. Optical coherence tomography (OCT) was performed using Phoenix Micron IV image-guided OCT system. Flash visual evoked potential (FVEP) and flash electroretinogram (FERG) and detection were performed using Roland ophthalmic electrophysiological diagnostic system.

### Synthesis of poly (PDSD-co-DDPD)-PEG (P1)

P1 were synthesized by condensation polymerization. In detail, mPEG5000OH, PDSD, DDPD were first dried under vacuum at 90 °C. PDSD (3.15 g, 12.5 mmol) was activated by CDI (4.46 g, 27.5 mmol) in dried DMF (30 mL) at room temperature (RT) until there was no bubble generation. The activated acid solution was dropwise added into the dried mPEG5000OH (1.25 g, 0.25 mmol) and DDPD (3.02 g, 12.5 mmol). The reaction was carried out at 60 °C under the protection of N_2_ for 72 hours (h). Subsequently, the reaction solution was concentrated, and the residual was dialyzed against deionized water for 72 h, and then the solution was transferred into a centrifuge tube and centrifuged for 5 min at the speed of 3000 rpm. Lastly, the supernatant was lyophilized to obtain P1.

### Nanoparticles formulation of MT-NPs

Melatonin (1 mg) and P1 (10 mg) were dissolved in THF to prepare an organic solution by bath sonication which was further filtrated through a 0.22 µm filter. Then, a 1 mL well-dispersed THF solution with melatonin and P1 was rapidly injected into the 9 mL distilled-deionized water. After that, the THF in the solution was dialyzed against water. The obtained aqueous solution containing melatonin was further filtered through a polyethersulfone (PES) filter (0.22 µm), which was subsequently washed and concentrated by ultracentrifugation at a speed of 3,500 rpm for 20 min. The concentration of melatonin solution was determined by ICP-MS.

### RI/R animal model construction

Healthy male Sprague-Dawley (SD) rats weighted 220-260 g were used in the study. Sodium pentobarbital was used in all rat surgeries (40mg/kg). To establish a rat model of retinal RI/R injury, the anterior chamber of the eye was punctured using a 30-gauge needle, followed by perfusion with sterile saline at a constant pressure of 110 mmHg for 60 min to induce transient ocular hypertension. At 12 h after RI/R, MT, NPs, and MT-NPs were administered via intravitreal injection at a volume of 4 μL per eye. All animal procedures were reviewed and approved by the Medical Ethics Committee of Xiangya Hospital, Central South University.

### Cell culture

R28 cell is an adherent retinal precursor cell line derived from postnatal day 6 SD rat retina immortalized with the 12S E1A gene, and has been used previously in studies on oxidative stress in retinal cells 57. Cells were cultured in DMEM low-glucose medium supplemented with 10% fetal bovine serum and 1% penicillin-streptomycin solution. The oxygen and glucose deprivation/reperfusion model (R28^OGD/R^) was established as followed. Briefly, the culture medium was replaced by glucose-free medium and R28 cells were cultured in the hypoxia incubator (1% O_2_) (v/v) for 4 h. Cells were then reoxygenated for another 12 h with normal medium and conditions until the experiment was conducted 58. Drug intervention was performed 4 h before modeling.

### Western blotting

Rat's retinas and R28 cells were collected and mixed with ice-cold cell lysates buffer (RIPA: cocktail inhibitor = 100:1), respectively. Subsequently, retinas and cells were homogenized and centrifuged. Stirred the supernatant with the loading buffer and heated the mixture at 100°C for 5 min. 30 μg protein sample of each group was loaded into SDS-PAGE gel and were separated by SDS-PAGE gel electrophoresis. Next the protein was transferred to the PVDF membrane, blocked with 5% non-fat milk for 2 h and incubated with BAX, cleaved-Caspase-3, cleaved-Caspase-7, RIPK1, RIPK3, p-RIPK3, p-MLKL, NLRP3, N-GSDMD and N-GSDME primary antibodies overnight. Then, the membrane was washed and followed with incubation of HRP-conjugated secondary antibodies for 1 h and detected by chemiluminescent reagent.

### Retinal section immunofluorescence

Retinal sections were blocked by incubation in 5% donkey serum containing 0.3% Triton X100 for 2 h at RT. To visualize the colocalization of PANoptosis-related proteins with RGCs, retinal sections were incubated with cleaved-Caspase-3 antibody (1:200), p-MLKL antibody (1:200), N-GSDMD antibody (1:200), and RBPMS antibody (1:200) in 5% donkey serum containing 0.1% Triton X-100 overnight at 4 °C, followed with Alexa Fluor 488/594 secondary fluorescent antibody (1:1000). Sections were then counterstained with DAPI for 5 min to visualize nuclei, followed by mounting with medium for imaging.

### ELISA

The levels of IL-1β in rat retinas and R28 cell culture supernatant were detected using the ELISA kits (Proteintech, Cat# KE20021). The supernatants from retina tissue homogenate and R28 cell culture were collected and analyzed according to the manufacturer's instructions.

### LDH release assay

LDH release from R28 cells was measured using a LDH release assay kit (Beyotime, #C0019S) according to the manufacturer's instructions. The LDH release rate of R28 cells was calculated as the percentage of the absorbance value: LDH release rate %= (experimental LDH - spontaneous LDH)/(maximum LDH release - spontaneous LDH) ×100.

### Caspase activity assay

Caspase-1 and Caspase-3 activity was measured using Caspase-1 activity assay kit (Beyotime, #C1101) and Caspase-3 activity assay kit (Beyotime, #C1115) according to the manufacturer's instructions.

### Antioxidant activity

The hydrogen peroxide (H_2_O_2_) scavenging activity of MT-NPs was evaluated with hydrogen peroxide detection kit. To begin with, MT, NPs and MT-NPs of various MT concentration (0.1 μM, 1 μM, 10 μM and 100 μM; Empty NPs at concentrations equivalent to those of MT-NPs) were incubated in 2 ml of PBS containing 50 mM H_2_O_2_ at RT for 1 h, or MT, NPs and MT-NPs (100 μM) were incubated in 2 ml of PBS containing 50 mM H_2_O_2_ at RT for 1 h, 2 h and 3 h. Then added 50 µL of samples or standard solution (1, 2, 5, 10, 20, 50 and 100 µM) to a 96-well plate. Next, 100 µL of hydrogen peroxide detection reagent was added to each well. After 30 min incubation, the concentration of residual H_2_O_2_ was measured by detecting the absorbance value at 560 nm with a Bio-Rad multiple plate reader, and the H_2_O_2_ scavenging ability was calculated. As for •ABTS^+^ assay, 10 μL MT, NPs and MT-NPs of various concentration (0.1 μM, 1 μM, 10 μM and 100 μM) were mixed with the 200 μL •ABTS^+^ working solution for 1 h, or 10 μL MT, NPs and MT-NPs (100 μM) were mixed with 200 μL •ABTS^+^ working solution for 1 h, 2 h or 3 h. Then the absorbance of the mixture was evaluated with a Bio-Rad multiple plate reader. All the operations were conducted at RT and in darkness.

### CCK-8 assay

R28 cells were treated with NPs, MT and MT-NPs at different MT concentrations ranging from 0.01 μM to 500 μM. R28^OGD/R^ model was founded after 4 h of drug intervention. CCK-8 solution was added to each well at 10 μL at the determined time. The plate was continuously incubated at 37°C for an additional 30 min. Subsequently, the OD-value at 450 nm was read by microplate reader.

### DCFH-DA/Hypoxyprobe/Calein AM-PI staining

For DCFH-DA staining, R28^OGD/R^ with diverse treatments were treated with 5 μM DCFH-DA in darkness for 20 min and washed with PBS. Nuclei were stained with Hoechst. For Hypoxyprobe staining, R28^OGD/R^ with diverse treatments were incubated with Hypoxyprobe in darkness for 60 min and washed with PBS. For Calcein AM/PI staining, R28^OGD/R^ with various treatments were co-stained with 5 µM PI and 10 µM Calcein-AM in darkness for 20 min at 37 °C. Then the cells were washed with PBS.

### Flow cytometry

After MT/NPs/MT-NPs treatment and OGD/R modeling, R28 cells were processed into a single-cell suspension and stained with Annexin V/PI or DCFH-DA according to the manufacturer's instruction. The cell samples were washed with PBS twice after staining with corresponding fluorescent dyes. The data was analyzed with Flowjo software.

### LC-MS detection

A high-purity MT standard was carefully weighed and dissolved in methanol to prepare a stock solution. Serial dilutions were prepared from the stock solution to obtain various concentrations of MT. Each concentration was analyzed using mass spectrometry under the same experimental conditions as the samples. The peak areas corresponding to each concentration were recorded, and a calibration curve was created by plotting the peak area against MT concentration. Linear regression analysis was performed to derive the equation of the standard curve, and the linearity was confirmed by calculating the correlation coefficient. This standard curve was then used to quantify the MT content in the experimental samples. Retinal samples were collected from rats with different treatments. The retinal samples were homogenized and centrifuged. The supernatant was collected and filtered through a 0.22 µm filter. The MT content in the retina was measured using a chromatograph and mass spectrometer according to the standard curve.

### Retinal whole-mount immunofluorescence

Retinal whole-mounts were blocked by 5% BSA containing 0.1% Triton X100 for 2 h at RT. To detect the RGC survival, retinas were incubated with anti-RBPMS antibody (1:400), followed by incubation of Alexa Fluor 594-conjugated donkey anti-rabbit secondary fluorescent antibody (1:1000). Retinal wholemounts were covered with a mounting medium. To assess the axon fasciculation density of RGCs, retinas were incubated with anti-pNF antibody (1:400), followed by incubation of Alexa Fluor 488-conjugated donkey anti-mouse secondary fluorescent antibody (1:1000). Retinal sectors with preserved axon fasciculation were selected in each retinal quadrant, a 20x field of view was selected at an eccentricity of 2000 μm from the ONH. To quantify the fascicles per view, a 200-μm-long line was traced perpendicular to the fascicles within the healthiest quadrant of each retina. The average number of pNF^+^ RGC axons per fascicle was counted along each line. Counts included axons bundled together in a fascicle, as well as defasciculated, single axons 59.

### Hematoxylin-eosin (H&E) staining

For histological examination, eyeballs of sacrificed rats with different treatments were fixed with 4% paraformaldehyde at RT for 24 h and washed with 70% alcohol. They were then embedded in paraffin and cut into sections (10 µm) in the vertical meridian through the optic disc. The sections were stained with hematoxylin and eosin after deparaffinized. The sections were scanned with a Pannoramic section scanner. The images were analyzed with CaseViewer to measure the ganglion cell complex thickness at a distance of 200 µm from the optic nerve.

### TUNEL assay

To evaluate apoptosis of RGCs in rat^RI/R^ after different treatments, we performed TUNEL assay kit. To labeled RGCs, retinal sections were incubated with anti-RBPMS antibody, followed by incubation of Alexa Fluor 594-conjugated donkey anti-rabbit secondary fluorescent antibody. Next, retinal sections were incubated with TUNEL solution at 37 °C for 1 h and covered with a mounting medium containing with DAPI. For quantification of RBPMS / TUNEL double positive cells, a 20x field of view was selected in the mid-peripheral region of each retinal section. The number of RBPMS / TUNEL double positive cells in each captured region were counted.

### Dihydroethidium (DHE) staining

DHE staining was conducted to measure retinal ROS production in rat^RI/R^ with different treatments. In brief, retinal sections were firstly fixed for 15 min with eyeball fixation solution, then washed with PBS for three times. The eye tissues were subsequently permeated utilizing 0.1% Triton X-100 and incubated with 5 μM DHE solution at 37 °C for 30 min.

### Optical coherence tomography (OCT) scanning

For OCT test, rats were anesthetized using 1% pentobarbital sodium and placed on an examiner. When the scan beam was perpendicular to the corneal surface, started OCT testing. Normal saline was administered to eyes every 2 min during the examination to keep the corneas lubricated. The optic nerve head was utilized as a landmark and located as the center while OCT images were taken. The images were taken at a distance of 200 µm from the optic nerve and were analyzed with Phoenix software to measure the ganglion cell complex thickness.

### FVEP

Rats with different treatments were anesthetized with 1% pentobarbital sodium and placed on a table with three recording electrodes inserted respectively under the skin of anterior bregma (cathode), occipital bone (anode), and ear (ground electrode). Unilateral FVEP data were obtained from each side of the eye in darkness. FVEP was performed using the Roland ophthalmic electrophysiological diagnostic system. The average latency and amplitudes of P2 wave for each group were counted and analyzed.

### FERG

Rats were absolutely dark adaptation for 24 h before FERG test. Then animals were anesthetized with 1% pentobarbital sodium and placed on the testing table. A low amount of 2.5% methylcellulose gel was smeared to each eye, and a copper loop electrode designed specifically for rats was placed on the cornea to record the ERGs. Needle reference and ground electrodes were inserted into the cheek and tail, respectively. Standard combined ERG (dark adapt 3.0 ERG) was recorded using Roland ophthalmic electrophysiological diagnostic system. The average amplitudes of a/b-wave for each group were counted and analyzed.

### Visual cliff test

A visual cliff apparatus consisted of a neat glass box in a dimension of 50 × 50 × 43 cm, divided into two rooms by a central platform, the shallow side with a checkered pattern inside, and the deep side with a same checkered pattern positioned 3 feet under it to create the illusion of depth. Rats were placed on the central platform, and their choices to step down were recorded. Each rat was subjected to the test once. The box and central platform were thoroughly cleaned after each test.

### Looming visual stimulus response test

Looming visual stimulus response test was performed in an enclosure with dimensions of 40 × 50 × 40 cm. A board was placed at one end of the enclosure at the height of 20 cm to act as a hideout. Food pieces were placed at the side opposite the hideout to encourage rats to explore their environment and remain outside of the hideout. A monitor was placed on top of the enclosure to display the looming stimulus, a video of an expanding black disk on a gray background made using Blender software. The stimulus was displayed 15 times, with a 500-ms interval between presentations. An overhead camera recorded rat behavior. Rats with various treatments were placed in the enclosure for 10 min prior to stimulus onset to allow time to acclimate. Three responses were assessed during the looming stimulus: freezing, fleeing, and tail rattling. If a rat demonstrated at least one of these behaviors over the course of the stimulus, it was tallied as a positive looming responder. Each rat was subjected to the test once. The enclosure was thoroughly cleaned after each test.

### Pupillary light reflex

Prior to pupillary light reflex recording, rats were subjected to a 2-hour dark adaptation period to ensure maximal pupillary dilation. Subsequently, the rats were lightly anesthetized and restrained on an appropriate rat holder. During the recording, bright light (1000 lx) was used to stimulate the rat's eye for 1 min. An infrared camera was positioned to the stimulated eye to record the pupil light reflex. The area of the pupils before and after light stimulation was measured by using ImageJ. The quantitative analysis was performed by comparing the reduced pupil area (pupil area before light stimulation minus pupil area after light stimulation) with the pupil area before light stimulation.

### Data analysis

All the results were presented as the means ± standard deviations (means ± SDs) of data from at least three independent experiments. Statistical analyses were performed on GraphPad Prism (version 8.0). Unpaired two-tailed Student t-test and one-way analysis of variance (ANOVA) followed by Tukey's multi-comparisons test was performed to test differences between groups. Statistically significant was defined at *p* < 0.05.

## Results and Discussion

### Acute high IOP induces PANoptosis activation in rat retinas

**Figure 1:**
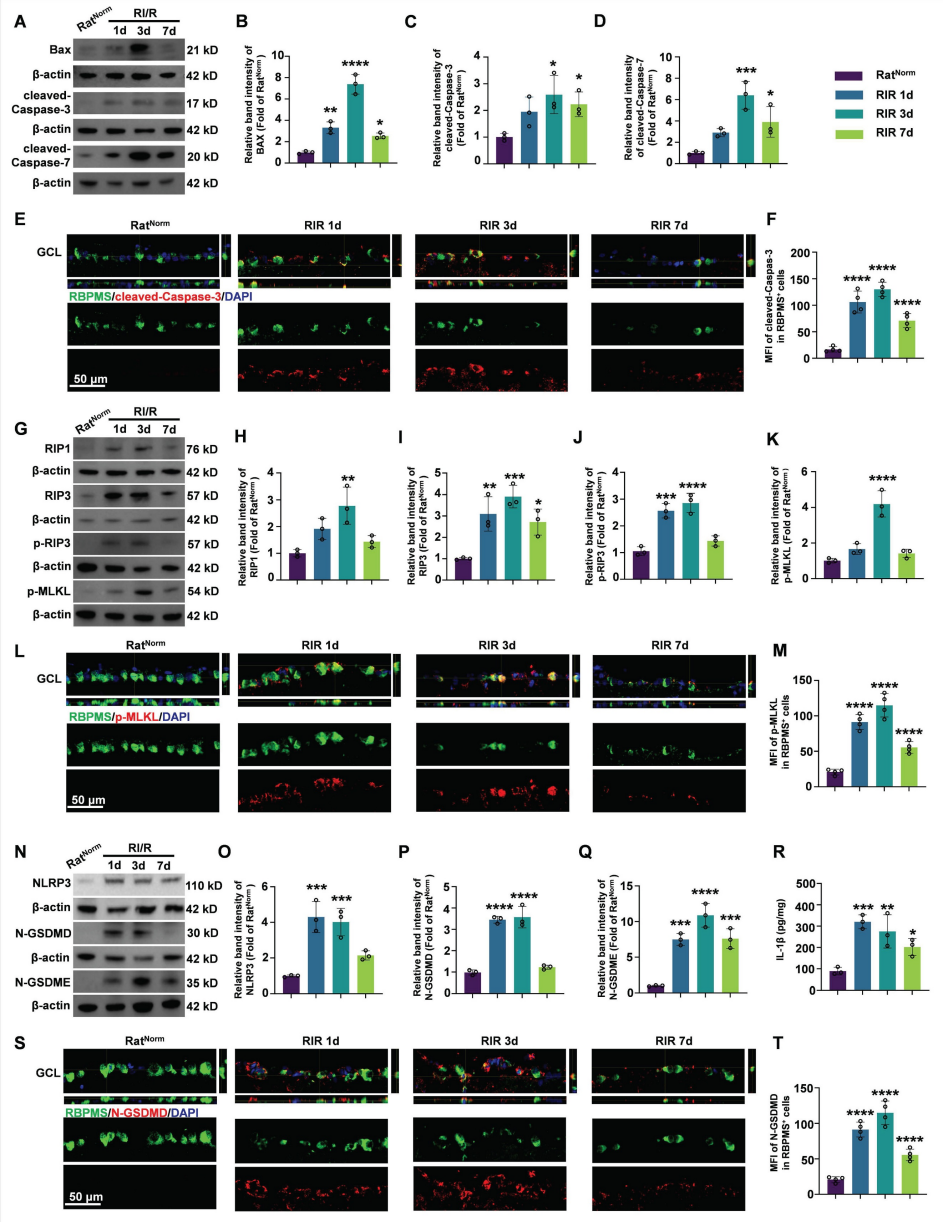
**Dynamics of PANoptosis activation in rat retinas after RI/R injury. A.** Western blotting showed the dynamic changes in apoptosis-related proteins level in rat retinas following RI/R injury.** B-D.** Relative band intensity of BAX (B), cleaved-Caspase-3 (C) and cleaved-Caspase-7 (D). **E.** Immunofluorescence images showed the colocalization of cleaved-Caspase-3 (red) with RBPMS (green)/DAPI (blue) in ganglion cell layer (GCL) of rat retinas following RI/R injury. **F.** Quantification of cleaved-Caspase-3 fluorescence intensity in RBPMS⁺ cells. **G.** Western blotting showed the dynamic changes in necroptosis-related proteins level in n rat retinas following RI/R injury. **H-K.** Relative band intensity of RIPK1 (H), RIPK3 (I), p-RIP3 (J) and p-MLKL (K). **L.** Immunofluorescence images showed the colocalization of p-MLKL (red) with RBPMS (green)/DAPI (blue) in GCL of rat retinas following RI/R injury. **M.** Quantification of p-MLKL fluorescence intensity in RBPMS⁺ cells. **N.** Western blotting showed the dynamic changes in pyroptosis-related proteins level in rat retinas following RI/R injury. **O-Q**. Relative band intensity of NLRP3 (O), N-GSDMD (P) and N-GSDME (Q). **P.** IL-1β levels in rat retinas following RI/R injury were determined by ELISA. **S.** Immunofluorescence images showed the colocalization of N-GSDMD (red) with RBPMS (green)/DAPI (blue) in GCL of rat retinas following RI/R injury. **T.** Quantification of N-GSDMD fluorescence in RBPMS⁺ cells. GCL: ganglion cell layer. Data are presented as the mean ± SD (n = 3-4 rats). **p < 0.01, ***p < 0.001, ****p < 0.0001 (compared with the Rat^Norm^ group using one-way analysis of variance followed by Tukeys post-hoc test).

Although a growing body of evidence supports the activation of PANoptotic pathways following acute glaucomatous injury 60, the dynamic patterns post-injury are not well characterized. To explored the dynamics of PANoptosis activation in acute glaucoma, we measured the protein levels of apoptotic, necroptotic, and pyroptotic hallmarks at multiple timepoints after RI/R injury. BAX, a pro-apoptotic protein, initiates the apoptotic cascade by inducing mitochondrial fragmentation and cytochrome c release, ultimately leading to cleavage and activation of Caspase-3/7. Cleaved-Caspase-3/7 are pivotal for initiating apoptosis via the cleavage of numerous target proteins 61. Western blotting revealed that the protein levels of BAX, cleaved-Caspase-3, and cleaved-Caspase-7 in the retina started to increase on day 1 after RI/R, peaked on day 3, and then declined by day 7 post-injury (Figure 1A-D). Subsequently, *in vivo* immunofluorescence demonstrated that the fluorescence intensity of cleaved-Caspase-3 staining in RBPMS⁺ RGCs increased continuously until day 3 post-injury, followed by a decrease (Figure 1E-F).

During necroptosis, RIP1 recruits RIP3 to form the RIP1/RIP3 complex. Following a series of auto/cross-phosphorylation events, phosphorylated-RIP3 (p-RIP3) phosphorylates and activates MLKL. Phosphorylated MLKL (p-MLKL) translocates to the plasma membrane, where it forms lytic pores and induces cell death 62, 63. Western blotting results showed that the protein levels of RIP1, RIP3, p-RIP3, and p-MLKL in the retina were significantly upregulated on day 1 post-injury, remained elevated until day 3, and then decreased thereafter (Figure 1G-K). Furthermore, double immunofluorescence assays revealed that the fluorescence intensity of p-MLKL staining significantly increased in RBPMS⁺ RGCs on day 1 and 3 post-injury, with a reduction observed on day 7 (Figure 1L-M).

In the pyroptosis pathway, NLRP3 forms an inflammasome with the adaptor protein apoptosis-associated speck-containing (ASC) and pro-Caspase-1. Pro-Caspase-1 is subsequently cleaved to form Caspase-1, which cleaves GSDMD. The N-terminal of GSDMD (N-GSDMD) forms pores in the plasma membrane, leading to cell death and secretion of IL-1β and IL-18 64-66. Furthermore, Caspase-3 cleaves GSDME and the N-terminal fragment of GSDME (N-GSDME) forms membrane pores to mediate pyroptosis 67. Our western blotting results showed that the protein levels of NLRP3, N-GSDMD, and N-GSDME in the retina were continuously elevated at 1 day and 3 days after RI/R, followed by a decrease (Figure 1N-Q). ELISA of IL-1β exhibited a similar pattern of changes. (Figure 1R). Immunofluorescence results revealed that the fluorescence intensity of N-GSDMD in RBPMS⁺ RGCs showed an initial increase on day 1, then a decrease on day 7 (Figure 1S-T).

We also detected dynamic changes in the expression of the hallmarks of PANoptosis in the OGD/R model of R28 cells *in vitro*. Our western blotting and ELISA results demonstrated that the protein levels of PANoptosis hallmarks and IL-1β were significantly upregulated at 6 h post-injury, peaked at 12 h, and then decreased at 24 h (Figure S1). Both necroptosis and pyroptosis lead to membrane pore formation and cell lysis accompanied by the release of lactate dehydrogenase (LDH). Therefore, the LDH released into the cell culture was used to quantify necroptosis and pyroptosis. The LDH release assay showed that LDH release began to increase at 6 h and peaked at 12 h after OGD/R (Figure S1J).

Subsequently, we assessed changes in retinal ROS levels after RI/R injury by conducting dihydroethidium (DHE) staining of retinal sections. Our results demonstrated a robust increase in DHE fluorescence intensity in the retina as early as day 1 post-RI/R injury. This elevation persisted until day 3 post-injury and partially declined by day 7 (Figure S2). These findings indicate an early and sustained increase in retinal ROS levels after RI/R injury.

Overall, these results revealed that the PANoptosis pathway was activated in RGCs early after RI/R injury and peaked at 3 days post-injury. Concomitantly, the retina exhibits early and sustained high ROS levels following RI/R injury. Mounting evidence indicates that ROS and PANoptosis promote each other 68. We therefore hypothesized that a positive feedback loop exists between ROS and the PANoptosis pathway in the retina following RI/R injury, which exacerbates RGC damage. Consequently, simultaneous scavenging of ROS and effective inhibition of PANoptosis are crucial for protecting RGCs following RI/R injury.

### MT-NPs synthesis and characterization

**Figure 2:**
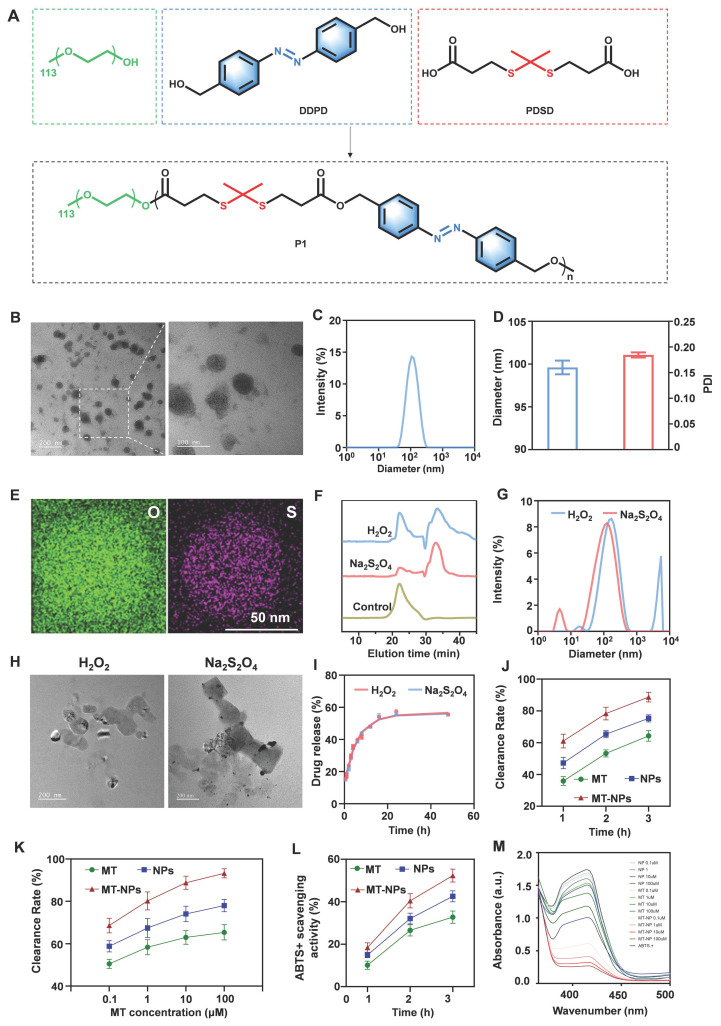
** Characterization of MT-NPs. A.** Schematic of P1 synthesis. **B.** Representative TEM images of MT-NPs. **C-D.** Hydrodynamic diameters and PDI of MT-NPs detected by DLS. **E.** Representative EDX images of MT-NPs. **F.** The change of dissociation curve of MT-NPs after incubation with H_2_O_2_ or Na_2_S_2_O_4_ for the indicated time. **G.** DLS determination of the particle size change of MT-NPs after H_2_O_2_ or Na_2_S_2_O_4_ treatment.** H.** Representative TEM images of MT-NPs after H_2_O_2_ or Na_2_S_2_O_4_ treatment. **I.** The release rate of MT from MT-NPs after H_2_O_2_ or Na_2_S_2_O_4_ treatment. **J.** Scavenging H_2_O_2_ by MT and MT-NPs at various time points. **K.** Scavenging H_2_O_2_ by MT and MT-NPs at different concentrations. **L.** Scavenging •ABTS^+^ by MT-NPs at various time points. **M.** Scavenging •ABTS^+^ by MT-NPs of various concentration. Data are presented as the mean ± SD (n = 4).

Rapid IOP elevation induces ischemia and severe hypoxic conditions in the retina 69. RGCs were extremely vulnerable to hypoxia, which leads to mitochondrial dysfunction, reduced ATP production, and excessive ROS generation 70. Therefore, developing an appropriate delivery system to achieve controlled drug release in RGCs under intracellular hypoxia and high ROS concentration is essential for acute glaucoma treatment. In the current study, we designed MT-NPs, which are ROS/hypoxia dual-responsive nanoparticles. Initially, an amphiphilic polymer Poly (PDSD-co-DDPD)-PEG (P1) was synthesized (Figure 2A; Figure S3) and characterized through nuclear magnetic resonance spectroscopy (1H-NMR; Figure S4). Next, P1 and MT were co-assembled to generate MT-NPs. Transmission electron microscopy (TEM) revealed that MT-NPs exhibited a uniform spherical nanostructure (Figure 2B). Dynamic light scattering (DLS) analysis confirmed that the average hydrodynamic diameter of MT-NPs was 99.6 nm, with a polydispersity index (PDI) of 0.18 (Figure 2C-D). Moreover, MT-NPs composition was further analyzed through scanning transmission electron microscopy (STEM)/energy dispersive X-ray spectroscopy (EDX). As depicted in Figure 2E, the EDX spectra of oxygen (O) and sulfur (S) elements could be observed. These results demonstrated that MT-NPs were effectively prepared.

To evaluate the dissociation ability of MT-NPs in an environment with excessive ROS and hypoxia, P1 was treated with H_2_O_2_ and sodium thiosulfate (Na_2_S_2_O_4_). H_2_O_2_ induced ROS accumulation, resulting in thioketal bond cleavage in P1. Na_2_S_2_O_4_ scavenged oxygen to simulate hypoxic conditions, leading to azo bond cleavage in P1. As illustrated in Figure 2F, gel permeation chromatography (GPC) revealed distribution of oligomeric units with lower molecular weight after treatment with both H_2_O_2_ and Na_2_S_2_O_4_. These results indicated that the P1 responded efficiently and degraded in a ROS/hypoxia environment, resulting in MT-NPs dissociation.

Next, MT-NPs' dissociation ability was investigated through DLS, and the results demonstrated that the MT-NPs particle size increased from 99.6 to 125.6 nm after 10 mM H_2_O_2_ treatment and to 169.9 nm after treatment with 10 mM Na_2_S_2_O_4_ (Figure 2G). The increased MT-NPs particle size after treatment with H_2_O_2_ and Na_2_S_2_O_4_ indicated their dual responsiveness of hypoxia and ROS. Furthermore, TEM images revealed that MT-NPs exhibited an aggregated, swollen morphology after incubation with H_2_O_2_ and Na_2_S_2_O_4_ (Figure 2H). Subsequently, we examined the MT release behavior of MT-NPs under H_2_O_2_ and Na_2_S_2_O_4_ treatment, and the results indicated a considerable increase in MT release rate in the presence of either H_2_O_2_ or Na_2_S_2_O_4_. Specifically, after 48 h of 10 mM H_2_O_2_ and Na_2_S_2_O_4_ treatment, the MT release rates were approximately 55.6% and 55.5%, respectively (Figure 2I), confirming the responsiveness of MT-NPs to ROS and hypoxia.

To evaluate their ROS clearance capability, we assessed the H_2_O_2_ and 2,2′-azino-bis (3-ethylbenzothiazoline-6-sulfonic acid) diammonium salt (•ABTS^+^) clearance rate of MT, empty NPs and MT-NPs 71-74. Figure 2J-M showed that both MT, empty NPs and MT-NPs scavenged H_2_O_2_ and •ABTS^+^ in a time- and dose-dependent manner. MT-NPs at 100 μM consumed approximately 89.5% of H_2_O_2_ and 54.7% of •ABTS^+^ when the incubation duration was 3 h. Taken together, these results confirmed that MT-NPs possess an effective ROS-scavenging ability, thereby facilitating the protection of RGCs from oxidative stress.

### Intracellular uptake, ROS scavenging and hypoxia alleviation of MT-NPs

**Figure 3:**
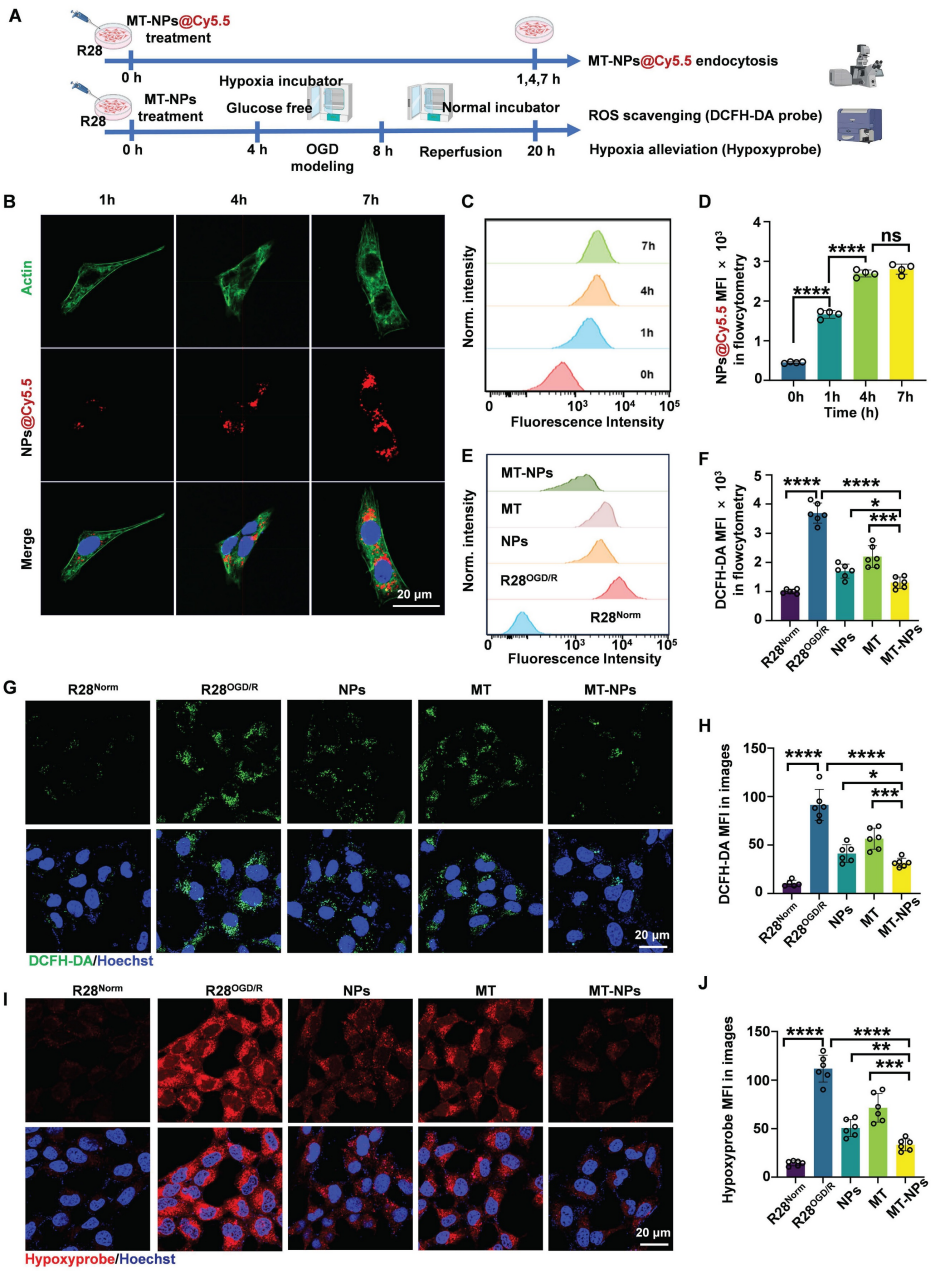
**Intracellular uptake, ROS scavenging and hypoxia alleviation by MT-NPs *in vitro*. A.** Schematic illustration of the intracellular uptake of MT-NPs, subsequent ROS scavenging and hypoxia alleviation in R28 cells. **B.** Representative images of R28 cells treated with MT-NPs@Cy5.5 at 1 h, 4 h and 7 h, respectively. The cell nucleus was stained by DAPI (blue). The red fluorescence came from Cy5.5. The cell skeleton was stained by Actin-Tracker Green-488 (green) respectively. **C, D.** Flow cytometry profiles (C) and quantification (D) of MT-NPs@Cy5.5 uptake at 0 h, 1 h, 4 h and 7 h. **E.** Flow cytometric detected ROS in R28 cells using DCFH-DA under different treatments. **F.** Quantification of mean fluorescence intensity (MFI) of ROS in R28 cells under different treatments. **G.** Representative images of R28 cells under different treatments. The green fluorescence came from DCFH-DA. The cell nucleus was stained by DAPI (blue). **H.** Quantification of ROS MFI in R28 cells under different treatments. **I.** Hypoxia was detected in R28 cells under different treatments. The red fluorescence came from Hypoxyprobe. The cell nucleus was stained by DAPI (blue). **J.** Quantification of Hypoxyprobe staining MFI in R28 cells under different treatments. Data are presented as the mean ± SD (n = 4-6), ns, not significant, *p < 0.05, **p < 0.01, ***p < 0.001, ****p < 0.0001 (comparisons between different groups in **D** were performed using one-way analysis of variance followed by Tukeys post-hoc test; comparisons between different groups in **F**, **H**, **J** were performed using Student t-test).

To assess MT-NPs uptake *in vitro*, we visualized and quantified the internalization of Cy5.5-labeled MT-NPs (MT-NPs@Cy5.5) in R28 cells using confocal microscopy and flow cytometry (Figure 3A). The results demonstrated a time-dependent increase in Cy5.5 fluorescence intensity in R28 cells, suggesting efficient uptake of MT-NPs (Figure 3B-D). However, the fluorescence intensity did not differ significantly between 4- and 7-h timepoints (Figure 3B-D). Recent studies have reported that after a period of rapid nanoparticle uptake by cells, the cellular endocytosis and exocytosis of nanoparticle may reach equilibrium, at which point the intracellular nanoparticle concentration remains relatively stable 75. Therefore, we selected 4 h for *in vitro* experiments with MT-NPs.

To assess the ability of MT-NPs to scavenge ROS and alleviate hypoxia in R28 cells subjected to OGD/R injury, we employed an ROS detection probe (DCFH-DA) and a hypoxia detection probe (Hypoxyprobe). The flow cytometry results showed that the DCFH-DA fluorescence intensity increased sharply in R28^OGD/R^. Treatment with empty NPs and MT reduced the DCFH-DA intensity to 46% and 59% of that in R28^OGD/R^, respectively. The DCFH-DA fluorescence intensity in MT-NPs-treated R28^OGD/R^ was further reduced to 35% of that in untreated R28^OGD/R^ (Figure 3E-F). The confocal imaging results of DCFH-DA staining (green) in each treatment group were consistent with the flow cytometry findings (Figure 3G-H). The fluorescence intensity of hypoxyprobe (red) positively correlated with the degree of hypoxia. Confocal images revealed that the hypoxyprobe staining intensity was substantially increased in R28^OGD/R^. Following MT treatment, this intensity was partially reduced to 64% of that in R28^OGD/R^, whereas it was 45% in the NPs-treated R28^OGD/R^. Notably, the hypoxyprobe staining intensity was significantly weaker in the MT-NP-treated group than that in the other treatment groups, reaching only 29% of that in R28^OGD/R^ (Figure 3I-J). These findings indicate that MT-NPs exert potent effects by scavenging ROS and alleviating hypoxia in OGD/R-injured R28 cells. This is likely a consequence of the integration of the ROS/hypoxia dual-responsive properties of NPs with the antioxidant capacity of MT-NPs.

### MT-NPs ameliorate OGD/R-induced pathological damage and death of R28 cells

**Figure 4:**
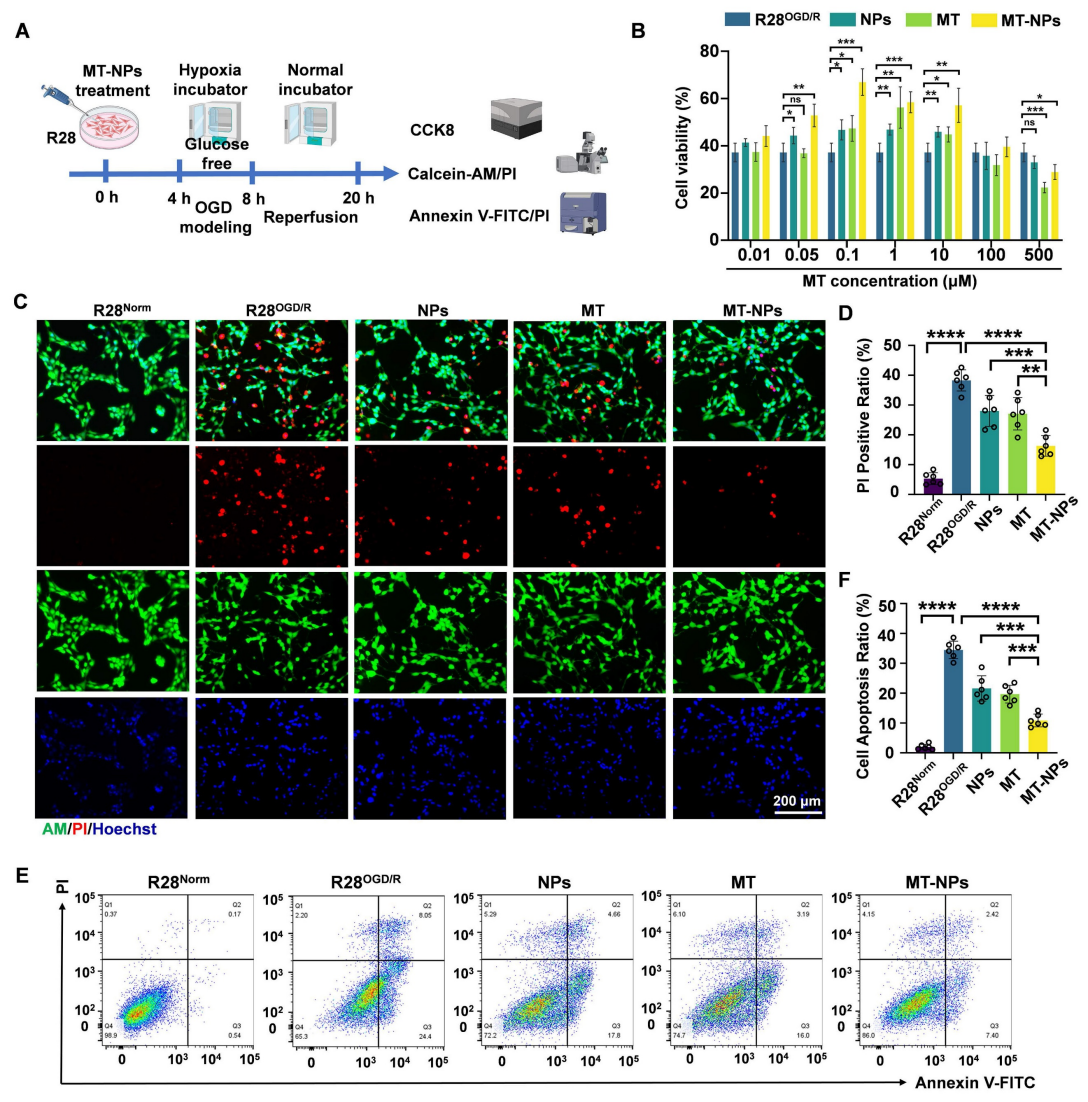
**MT-NPs alleviate OGD/R-induced R28 cell death. A.** Schematic illustration of MT-NPs-mediated protection of R28^OGD/R^ from pathological damage and death. The R28 cells were pretreated with MT-NPs for 4 h, and then a model of R28^OGD/R^ was constructed. CCK-8 and apoptosis assays were performed 12 h post-injury. **B.** CCK-8 assay assessing protection by NPs, MT and MT-NPs at different concentrations. **C.** Representative images of Calcein-AM (live, green) and PI (dead, red) staining under different treatments. **D.** Quantification of PI-positive cells. **E, F.** Flow cytometric profiles (E) and corresponding quantification (F) of apoptotic rates in R28 cells under different treatments. Data are presented as the mean ± SD (n = 6). ns, not significant, **p < 0.01, ***p < 0.001, ****p < 0.0001 (comparisons between different groups were performed using Student t-test).

Next, we examined whether MT-NPs effectively protected R28 cells from OGD/R injury. The cell counting kit 8 (CCK-8) assay, Calcein AM/PI staining, and annexin V-FITC/PI assay were used to detect the protective effects of MT-NPs (Figure 4A).

We first performed a CCK-8 assay to determine the optimal MT-NPs concentration for protecting R28 cells from OGD/R injury. The results revealed that the protective concentration range of MT is 0.1 to 10 μM. MT-NPs exerted a protective effect at MT concentrations ranging from 0.05 to 10 μM, and within this concentration range, empty NPs also afforded protection to R28^OGD/R^. However, we observed that MT exerted an inhibitory effect on R28 cells survival at higher concentrations (100 μM and 500 μM). Previous studies have reported that high concentrations of MT can induce cellular calcium overload and oxidative stress 76. Notably, at concentrations of 0.1 to 10 μM, MT-NPs exhibited stronger protective effects than MT, with the maximum efficacy observed at 0.1 μM (Figure 4B). Consequently, we used 0.1 μM as the intervention concentration for subsequent experimentation.

Calcein AM/PI staining was used to visualize the protective effects of MT-NPs against OGD/R-injury in R28 cells. Calcein AM permeates intact live cells and emits green fluorescence, whereas PI binds only to the DNA within dead cells and emits red fluorescence. The results demonstrated that NPs and MT reduced the proportion of PI-positive cells to 28% and 27%, respectively, whereas MT-NPs further diminished this proportion to 16% (Figure 4C-D). The Annexin V-FITC/PI assay was employed to further elucidate the effects of MT-NPs on apoptosis in R28 cells following OGD/R. Flow cytometry results showed that, compared with the 36% apoptotic rate in R28^OGD/R^, empty NP- and MT-treated groups exhibited reduced apoptotic rates of 22% and 19%, respectively. Notably, MT-NPs further decreased this rate to 9% (Figure 4E-F).

Overall, our evidence indicates that loading MT into NPs yields a robust synergistic effect, providing superior protection to OGD/R-injured R28 cells compared to MT and empty NPs.

### MT-NPs inhibit OGD/R-induced PANoptosis activation in R28 cells

**Figure 5:**
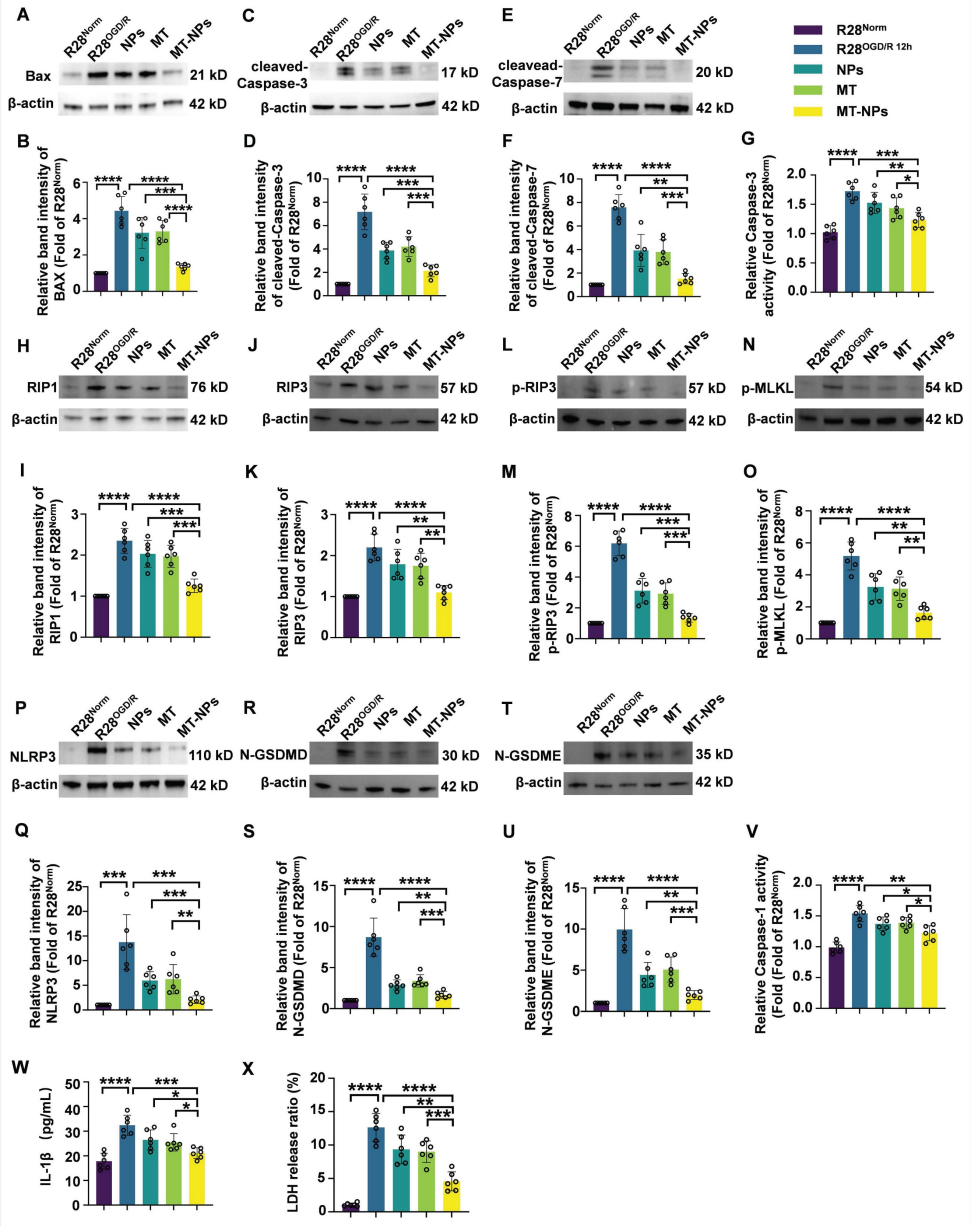
** MT-NPs inhibit OGD/R-induced PANoptosis in R28 cells. A, C, E.** Western blotting showed the changes in apoptosis-related proteins level in R28 cells under different treatments. **B, D, F.** Relative band intensity of BAX (B), cleaved-Caspase-3 (D) and cleaved-Caspase-7 (F). **G.** Relative Caspase-3 activity in R28 cells under different treatments.** H, J, L, N.** Western blotting showed the changes in necroptosis-related proteins level in R28 cells under different treatments. **I, K, M, O.** Relative band intensity of RIPK1 (I), RIP3 (K), p-RIP3 (M) and p-MLKL (O). **P, R, T.** Western blotting showed the changes in pyroptosis-related proteins level in R28 cells with different treatments. **Q, S, U.** Relative band intensity of NLRP3 (Q), N-GSDMD (S) and N-GSDME (U). **V.** Relative Caspase-1 activity in R28 cells under different treatments. **W.** IL-1β levels in R28 cells under different treatments were determined by ELISA. **X.** LDH release ratio in R28 cells under different treatments. Data are presented as the mean ± SD (n = 6). *p < 0.05, **p < 0.01, ***p < 0.001, ****p < 0.0001 (comparisons between different groups were performed using Student t-test).

Next, we explore the inhibitory effects of MT-NPs on PANoptosis in the R28^OGD/R^. Western blotting revealed that the OGD/R-induced increases in the protein levels of apoptosis (BAX, cleaved-Caspase-3, cleaved-Caspase-7) (Figure 5A-F), necroptosis (RIP1, RIP3, p-RIP3, p-MLKL) (Figure 5H-O) and pyroptosis (NLRP3, N-GSDMD, N-GSDME) (Figure 5P-U) hallmarks were partially attenuated after empty NPs and MT treatment. Notably, the reduction in the protein levels of these PANoptosis molecules was more pronounced in the MT-NPs group. Compared to empty NPs and MT, MT-NPs exerted the strongest inhibitory effect on Caspase-3 and Caspase-1 activity (Figure 5G, 5V). Furthermore, our results showed that empty NPs and MT could partially suppressed IL-1β secretion and LDH release, while in comparison, MT-NPs exhibited a more significant effect (Figure 5W-X).

The results in this section demonstrate that loading MT into NPs achieves a superior effect in inhibiting PANoptosis compared with empty NPs and free MT. Based on these *in vitro* data, we hypothesize that MT-NPs represent a viable therapeutic option for protecting RGCs against acute high IOP-induced RI/R injury.

### MT-NPs protect RGCs from RI/R injury

Acute glaucoma is characterized by a rapid decline in visual function. Therefore, timely and appropriate treatment of acute glaucoma is critical for preventing vision loss. Most previous studies investigating the neuroprotective effects of MT on RGCs under hypoxia- or ischemia-induced retinopathy employed repeated intraperitoneal administration for MT delivery 53, 54, 77. This therapeutic approach has two key limitations: 1) Systemic administration may not achieve effective MT concentrations in the retina quickly enough, and 2) MT itself exhibits a short plasma half-life (<30 min) 78, 79. Therefore, localized and controlled delivery strategies for MT in acute glaucoma should be developed.

**Figure 6:**
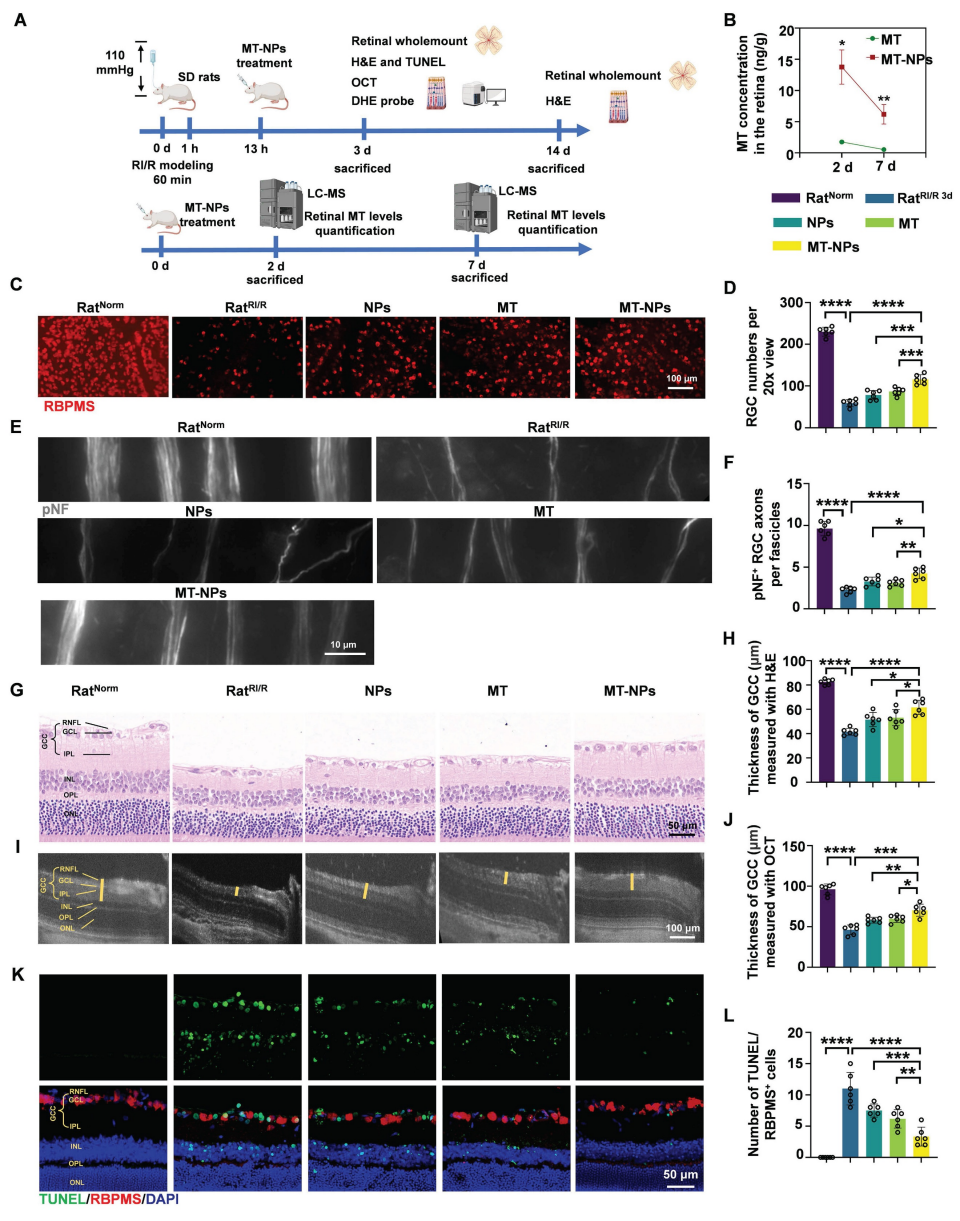
**MT-NPs protect RGCs from RI/R injury. A.** MT-NPs were intravitreally injected 12 h after the induction of RI/R injury in rats. On the 3rd day after RI/R construction, rat retinas were scanned by OCT, and then rats were sacrificed and the retinas were collected for H&E and IF. Retinal MT levels were quantified by LC-MS on the day 2 and day 7 after intravitreal injection. **B.** Retinal MT levels quantified by LC-MS at days 2 and 7 post-intravitreal injection. **C.** RBPMS labeled RGCs in the retinal whole-mounts under different treatments. **D.** Quantification of RGCs numbers per 20x view. **E.** pNF-labeled RGCs axons under different treatments. **F.** The number of pNF^+^ RGC axons per fascicle in each group. **G.** H&E staining images of rat retinas under different treatments. **H.** Quantification of GCC thickness via H&E staining under different treatments. **I.** OCT images of rat retinas under different treatments. **J.** Quantification of GCC thickness via OCT under different treatments. **K.** TUNEL assay of rat retinas under different treatments. **L.** The number of TUNEL (green)/RBPMS (red) double positive cells in rat retinas under different treatments. The cell nucleus was stained by DAPI (blue). Data are presented as the mean ± SD (n = 6 rats). ns, not significant, *p < 0.05, **p < 0.01, ***p < 0.001, ****p < 0.0001. RNFL: retinal nerve fiber layer, GCL: ganglion cell layer, IPL: inner plexiform layer, INL: inner nuclear layer, OPL: outer plexiform layer, ONL: outer nuclear layer (comparisons between different groups were performed using Student t-test).

Here, we first injected MT-NPs@Cy5.5 intravitreally; this administration method involves direct drug delivery into the vitreous cavity, which is most commonly used for treating retinal diseases 80. Following intravitreal injection of MT-NPs@Cy5.5, we assessed the internalization of MT-NPs in retinal cells to evaluate their biocompatibility. At day 2 after injection, retinal whole-mount demonstrated considerable accumulation of MT-NPs@Cy5.5, with robust fluorescence persisting until day 7 post-injection (Figure S7). Liquid chromatography-mass spectrometry (LC-MS) analysis revealed that, at day 2 and 7 after the intravitreal injections, the retinal MT concentration in the MT-NP-treated group was approximately 10-fold higher than that in the MT-treated group (Figure 6B). This finding suggests that NPs facilitate the prolonged local retention and controlled release of MT. Furthermore, hematoxylin-eosin (H&E) staining confirmed that intravitreal injection of MT-NPs did not damage the retina or other vital organs, indicating a favorable safety profile (Figure S8). These findings indicate that MT-NPs are effectively taken up by the retinal cells and are biologically safe.

Next, we assessed the protective effects of MT-NPs upon RGCs *in vivo*. MT, empty NPs and MT-NPs were intravitreally administered 12 h post-RI/R injury. Retinal whole-mount staining for RGC somas and axons, H&E staining, OCT scanning, TUNEL assay, and DHE staining (for ROS detection) were performed (Figure 6A).

To quantify the protective ability of MT-NPs in RGCs, we labeled RGC somas with RBPMS and counted the number of RBPMS-positive cells. We found that MT-NPs reduced RGC loss within the MT concentration range of 5 to 100 μM. Empty NPs at concentrations equivalent to those of MT-NPs, also exhibited a slight protective effect. The results showed that MT-NPs conferred superior protective efficacy compared with that of MT, with the maximal effect observed at 50 μM, which produced a 1.9-fold increase in the number of RBPMS-positive cells compared with the untreated Rat^RI/R^ (Figure 6C-D, Figure S9). Therefore, we set 50 µM as the intervention concentration for subsequent *in vivo* experiments. Progressive RGC loss has been reported in rodent RI/R models 81. Accordingly, we extended the assessment of RGC survival up to 14 days post-RI/R injury. The results showed that the number of RGCs decreased further at day 14 post-injury compared with that at day 3. At this time point, MT-NPs remained superior to MT alone and empty NPs in mitigating RGC loss, with the RGC count being approximately 2-fold that observed in the untreated Rat^RI/R^ (Figure S10).

In glaucoma, high IOP initially compresses RGC axons at the lamina cribrosa, leading to axonal swelling and axoplasmic flow blockage 82. Therefore, axonal degeneration is the earliest sign of RGC damage in acute glaucoma. To quantify the protective effects of MT-NPs in RGC axons, we assessed the integrity of RGC axon fasciculation by using phosphorylated neurofilament (pNF), a widely used cytoskeletal marker for tracking axons 57, 83, 84. Healthy retinas (Rat^Norm^ group) displayed axons uniformly segregated in tightly packed fascicles, whereas retinas at day 3 post-RI/R injury exhibited defasciculation, with fascicle thinning and solitary axons (Figure 6E). Statistical analysis revealed no significant difference in the number of fascicles per view among the groups (Figure S11). However, the Rat^RI/R^ group exhibited significantly fewer pNF^+^ RGC axons per fascicle on day 3 days post-injury, which was partially restored by empty NPs (~2.95) and MT and (~3.1). Remarkably, MT-NPs-treated Rat^RI/R^ displayed a considerable increase in the number of pNF^+^ RGC axons per fascicle (~4.42) (Figure 6F). These results confirmed that MT-NPs effectively preserved RGC axon fasciculation integrity following RI/R injury.

Alterations in the retinal ganglion cell complex (GCC) thickness reflect structural variation in RGC somas, axons, dendritic arbors, and synapses 85, representing a reliable glaucomatous damage biomarker 86. We used H&E staining and *in vivo* OCT imaging (for high-resolution in vivo retina visualization) 87, 88 to evaluate GCC thickness. The H&E staining results revealed that the GCC thickness was significantly reduced at day 3 post-RI/R injury (~41.6 µm). Treatments with empty NPs and MT restored GCC thickness to 51.6 µm and 53.1 µm, respectively. MT-NPs restored GCC thickness further to 61.6 µm—approximately 1.5 times that in untreated Rat^RI/R^ (Figure 6G-H). On day 14 post-RI/R injury, the GCC thickness continued to decrease (~23.2 µm). At this time point, we observed that GCC thickness showed a degree of restoration following intervention with empty NPs and MT. Compared to NPs and MT, MT-NPs exerted a more significant effect on the restoration of GCC thickness (Figure S12). The results of *in vivo* OCT scanning at day 3 post-injury corroborated the H&E findings, supporting the conclusion that MT-NPs potently restored the GCC thickness (Figure 6I-J). Overall, these results demonstrated that MT-NPs can effectively protect the structure of RGC somas, axons, dendritic arbors, and synapses after RI/R injury.

Subsequently, TUNEL (green)/RBPMS (red) double-staining was performed to evaluate RGC apoptosis after RI/R injury. Our results illustrated that the number of TUNEL/RBPMS double-positive (orange) cells was substantially higher in Rat^RI/R^ (~12) than in Rat^Norm^ (~0.33). This number was lower in empty NPs (~7.5) and MT-treated Rat^RI/R^ (~6.2). MT-NPs-treated Rat^RI/R^ (~3.1) showed a further decrease, reaching only 25% of that in the untreated Rat^RI/R^ (Figure 6K-L). Moreover, the MT-NPs treatment significantly reduced the number of apoptotic cells in the inner nuclear layer (INL) and outer nuclear layer (ONL) (Figure 6K). These results indicated that MT-NPs effectively suppressed apoptosis in RGCs and other retinal cells after RI/R injury.

We subsequently used the ROS probe, DHE, to investigate whether MT-NPs could scavenge retinal ROS after RI/R injury. As mentioned earlier, the fluorescence intensity of DHE staining showed a sustained increase on day 1 and 3 post-RI/R injury, reflecting an early and persistent elevation of ROS levels post-injury. Our results demonstrated, by day 3, treatment with empty NPs, MT, and MT-NPs reduced the DHE fluorescence intensity in Rat^RI/R^ by 54%, 25%, and 72%, respectively (Figure S13). These results indicate that the combination of MT and NPs achieved an efficient and sustained ROS-scavenging effect.

Overall, in this section, we clearly demonstrated that MT-NPs exhibit a more pronounced protective effect on RGCs than MT alone. This could be attributed to the ROS-scavenging capacity of NPs and their ability to enhance retinal retention of MT.

### MT-NPs inhibits PANoptosis activation *in vivo*

**Figure 7:**
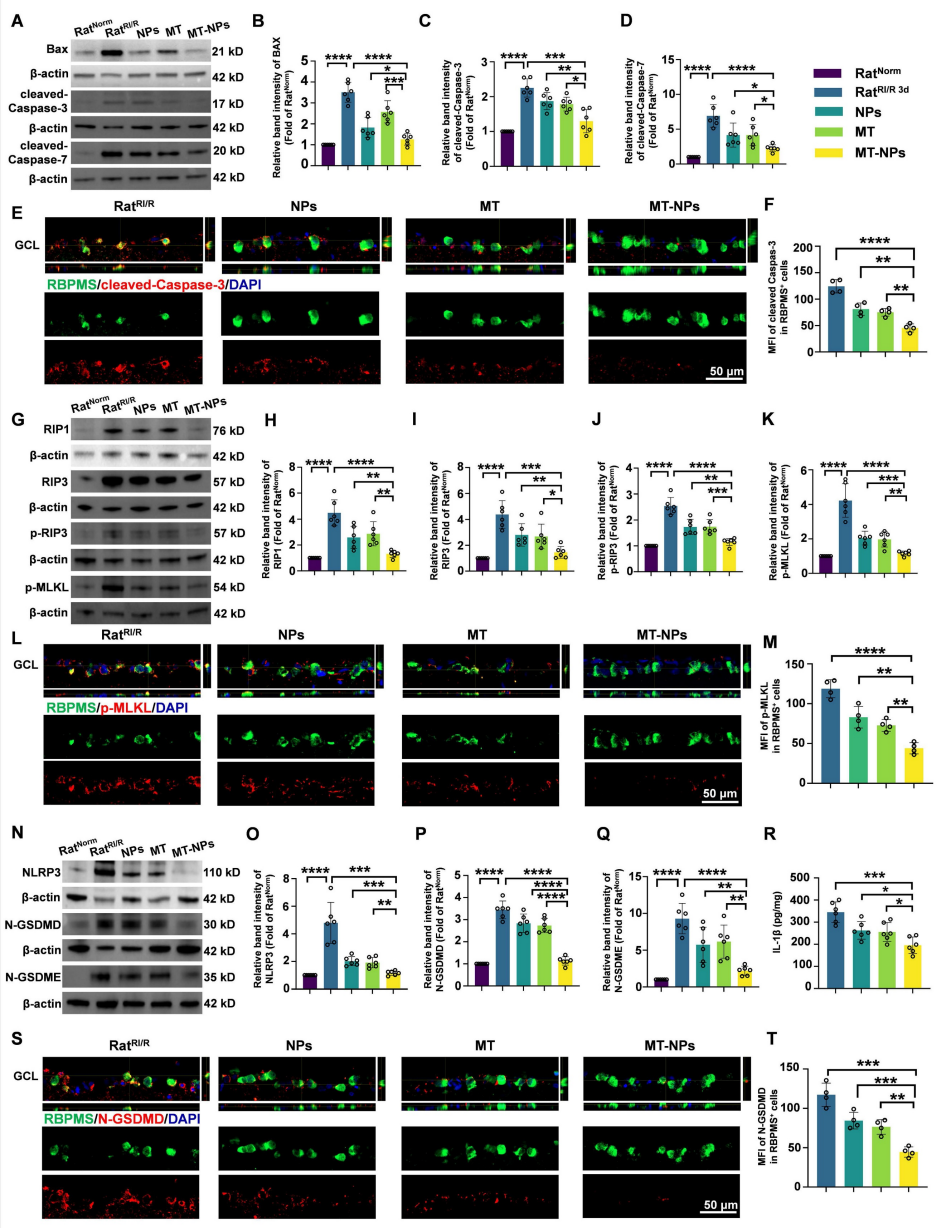
** MT-NPs inhibit RI/R-induced PANoptosis in rat ratinas. A.** Western blotting showed apoptosis-related proteins level in rat retinas under different treatments. **B-D.** Relative band intensity of BAX (B), cleaved-Caspase-3 (C) and cleaved-Caspase-7 (D). **E.** Immunofluorescence images showed the colocalization of cleaved-Caspase-3 (red) with RBPMS (green) in GCL of rat retinas under different treatments.** F.** Quantification of cleaved-Caspase-3 fluorescence intensity in RBPMS⁺ cells. **G.** Western blotting showed necroptosis-related proteins level in rat retinas under different treatments. **H-K.** Relative band intensity of RIPK1 (H), RIPK3 (I), p-RIP3 (J) and p-MLKL (K). **L.** Immunofluorescence images showed the colocalization of p-MLKL (red) with RBPMS (green) in GCL of rat retinas following RI/R injury.** M.** Quantification of p-MLKL fluorescence in RBPMS⁺ cells. **N.** Western blotting showed pyroptosis-related proteins level in rat retinas under different treatments. **O-Q**. Relative Band intensity of NLRP3 (O), N-GSDMD (P) and N-GSDME (Q). **R**. IL-1β levels in rat retinas following RI/R were determined by ELISA. **S**. Immunofluorescence images showed the colocalization of N-GSDMD (red) with RBPMS (green) in GCL of rat retinas following RI/R injury. **T.** Quantification of N-GSDMD fluorescence in RBPMS⁺ cells. GCL: ganglion cell layer. Data are presented as the mean ± SD (n = 4-6 rats). *p < 0.05, **p < 0.01, ***p < 0.001, ****p < 0.0001 (comparisons between different groups were performed using Student t-test).

We further investigated the *in vivo* inhibitory effect of MT-NPs on PANoptosis. Western blotting results showed that on day 3 after RI/R injury, both empty NPs and MT interventions partially reduced the expression of PANoptosis-related proteins. MT-NPs exhibited a more significant inhibitory effect on the expression of PANoptosis hallmarks (Figure 7A-D, G-K, N-Q). ELISA assay demonstrated that MT-NPs reduced IL-1β in the retina more intensely than other treatments (Figure 7R). Double immunofluorescence results displayed that, compared to that of the empty NP and MT groups, the reduction in the fluorescence intensity of cleaved-Caspase-3, p-MLKL, and N-GSDMD in RBPMS-labeled RGCs was more pronounced in the MT-NPs group (Figure 7E-F, L-M, S-T). In summary, our results indicated that the intravitreal injection of empty NPs or MT exerted an inhibitory effect on the activation of PANoptosis; however, when MT was loaded into the NPs, a more significant inhibitory effect on PANoptosis was achieved.

### MT-NPs preserve visual function and vision of rats after RI/R injury

**Figure 8:**
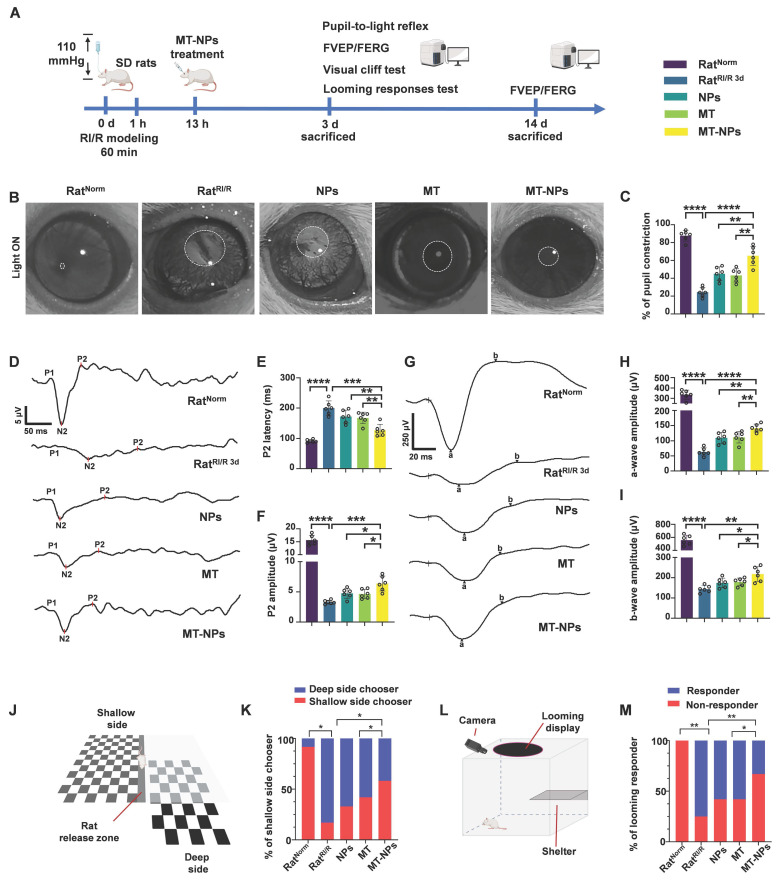
** MT-NPs rescue visual function and vision of rats following RI/R. A.** Visual function was evaluated on day 3 post-injury using pupil light reflex and vision-based behavioral tests. FERG and FVEP were recorded on day 3 and 14 after RI/R construction.** B.** Pupil light reflex results of rats under different treatments.** C.** Quantification of pupil restriction of rats under various treatments.** D.** The FVEP results of rats with various treatments. **E.** Quantification of FVEP P2 latency. **F.** The quantification of FVEP P2 amplitude. **G.** The FERG results of rats under various treatment. **H.** Quantification of FERG a-wave amplitude. **I.** Quantification of FERG b-wave amplitude. **J.** Visual cliff tests of rats with diverse treatments. **K.** Percentage of shallow side chooser with diverse treatments. **L.** Looming experiment of rats after various treatments. **M.** Percentage of looming responder in looming experiment after various treatments. Data are presented as the mean ± SD (n = 6 rats in pupil light reflex, FVEP and FERG; n = 12 rats in visual cliff tests and looming experiment). *p < 0.05, **p < 0.01, ***p < 0.001, ****p < 0.0001 (comparisons between different groups were performed using Student t-test).

Inspired by the protective effects of MT-NPs on RGCs both *in vitro* and *in vivo*, we investigated their effects on visual function (Figure 8A). We first examined the pupillary light reflex, reflecting the ability of RGCs to convey retinal information from the eye to the brain, thereby determining the integrity of electrical conduction activity between neurons in the visual system 89, 90. Under physiological conditions, abrupt exposure of the eye to light stimuli elicits pupillary constriction, a response that serves to adapt to the sudden increase in luminance. Conversely, impairment of the visual system can lead to delayed or even absent pupillary reflexes, which in turn results in pupillary dilation. In Rat^Norm^ group, light stimulation induced pupil constriction to 90%. However, RI/R caused a reduction of pupil constriction to 20 % on day 3 after injury. Interventions with NPs and MT restored pupil constriction to 45% and 55%, respectively, whereas the pupil constriction in the MT-NPs group was further increased to 70% (Figure 8B-C).

We next assessed visual pathway integrity based on flash visual evoked potential (FVEP) 91. The latency and amplitude of FVEP reflect the velocity of the signal along the visual pathway and the axonal degeneration of RGCs, respectively 91, 92. The FVEP results demonstrated that the P2 latency was significantly prolonged and the wave amplitude was reduced on day 3 post-RI/R injury. Empty NPs and MT treatment partially restored RI/R-induced prolonged latency and reduction in amplitude of P2. MT-NPs, however, restored the P2 latency and amplitude more significantly (Figure 8D-F). On day 14 post-injury, we observed a mild rescue effect of empty NPs and MT on P2 latency and amplitude. (Figure S14A-C). Notably, MT-NPs continued to exhibit a superior effect compared with that of NPs and MT. The FVEP results indicate that the protective effect of MT-NPs on the axonal structure of RGCs (Figure 6E) is effectively translated into the preservation of visual electrical signal conduction.

Next, we investigated whether MT-NPs protect the function of the entire retina using flash electroretinography (FERG) 93, 94. The amplitude of the FERG a-wave reflects the functional integrity of cone and rod photoreceptors. The amplitude of the b-wave is indicative of the functional status of bipolar cells and Müller cells. Our results showed that the amplitudes of the FERG a-wave and b-wave decreased significantly on day 3 and day 14 after RI/R injury. Consistent with the FVEP results, MT-NPs exhibited a more potent rescue effect on the amplitudes of FERG a-wave and b-wave on both day 3 and day 14 after injury compared with empty NPs or MT alone (Figure 8G-I, Figure S14D-F).

To determine whether MT-NPs-mediated visual pathway protection can preserve vision, we performed two vision-based behavioral tests. First, we conducted a visual cliff test to evaluate the ability of the rats to discriminate visual depth. This test is based on the innate tendency of rats to avoid the deep side of the visual cliff and step on the shallow side 95. Twelve rats in each group were placed on a central platform between the deep and shallow sides of the cliff and their choices toward either the deep or the shallow side were recorded. Our results showed that 11 rats in control group chose the shallow side, and this number decreased to 2 after RI/R injury. In the NPs and MT groups, 4 and 5 rats chose the shallow side, respectively, while in the MT-NPs group, this number increased to 7 (Figure 8J-K).

Second, a looming experiment was conducted on 12 rats per group in a box surrounded by an overhead monitor that continuously displayed looming stimuli to assess the innate defensive responses in rats. The rats were provided with a shelter inside the box with a camera to record their behavior. Rats with normal vision hid under the shelter, wagged their tails, or shivered when confronted with looming stimuli. We observed that all-12 Rat^Norm^ responded to looming stimuli, whereas only 3 Rat^RI/R^ responded. The numbers of rats responding to looming stimuli in the NPs, MT, and MT-NPs groups were 5, 5, and 8, respectively (Figure 8L-M).

Taken together, these findings suggested that MT-NPs could preserve vision effectively, transforming the RI/R-induced irregular activity pattern into a physiological state.

## Conclusions

In summary, we developed a ROS/hypoxia dual-responsive, biodegradable polymer containing azo and thioketal bonds, designed to encapsulate MT in NPs for intraocular delivery. This simplified and effective design significantly enhancing the biocompatibility and long-term retention of MT, thereby improving its clinical applicability. Notably, MT-NPs effectively alleviated hypoxia, cleared excess ROS, and inhibited PANoptosis in RGCs during acute glaucomatous injury. The results demonstrated that MT-NPs not only protect RGC axons and somas but also restore visual function in rats with acute glaucoma. Our study highlighted the potential of MT-NPs as an effective nanomedicine for acute glaucoma treatment and provided implications for the development of nanodrug delivery systems to treat other neurodegenerative conditions.

## Supplementary Material

Supplementary figures and table.

## Abbreviations

IOP: intraocular pressure
RGCs: retinal ganglion cells
APACG: acute primary angle-closure glaucoma
MT: melatonin
NPs: nanoparticles
ROS: reactive oxygen species
RI/R: retinal ischemia/reperfusion
OGD/R: oxygen-glucose deprivation/reperfusion
RNFL: retinal nerve fiber layer
GCL: ganglion cell layer
IPL: inner plexiform layer
INL: inner nuclear layer
OPL: outer plexiform layer
ONL: outer nuclear layer
OCT: optical coherence tomography
FVEP: flash visual evoked potential
FERG: flash electroretinogram
