# Supplementary material for: Reactive oxygen species/hypoxia dual-responsive polymers combined with melatonin inhibited PANoptosis of retinal ganglion cells for acute glaucoma treatment
Source: Theranostics. 2026 Jan 1;16(2):830–51. doi: 10.7150/thno.112836 (PMC12674935; doi:10.7150/thno.112836)
Supplement: Supplementary file 1 — Supplementary figures and table. [file thnov16p0830s1.pdf]

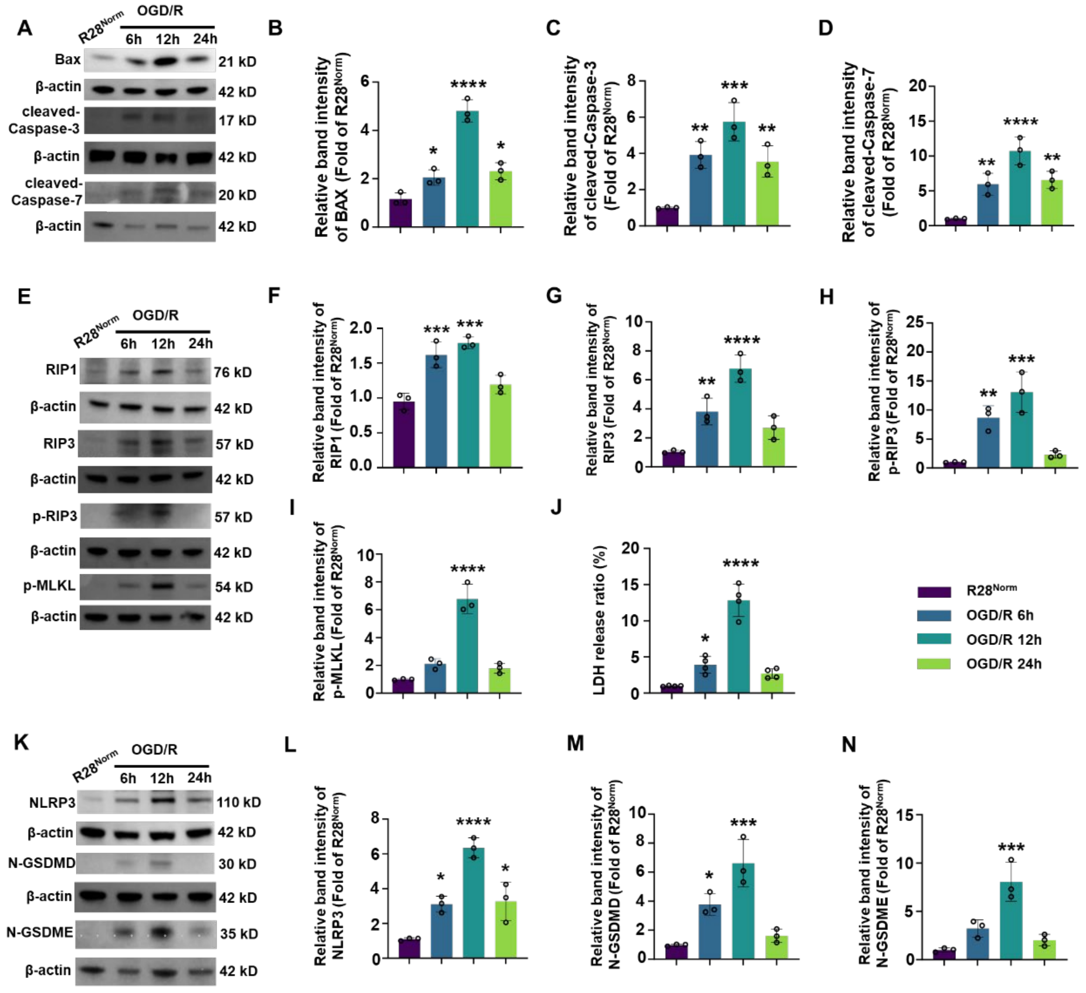

Figure S1. OGD/R induces PANoptosis activation in R28 cells. A. Western blotting showed the dynamic changes in apoptosis-related protein levels in R28 cells following OGD/R. **B-D.** Relative band intensity of BAX (B), cleaved-Caspase-3 (C) and cleaved-Caspase-7 (D). **E.** Western blotting showed the dynamic changes in necroptosis-related protein levels in R28 cells following OGD/R. **F-I.** Relative band intensity of RIP1 (F), RIP3 (G), p-RIP3 (H) and p-MLKL (I). **J.** LDH release ratio in R28 cells under different treatments were determined by LDH release assay. **K.** Western blotting showed the dynamic changes in pyroptosis-related protein levels in R28 cells following OGD/R. **L-N.** Relative band intensity of NLRP3 (L), N-GSDMD (M) and N-GSDME (N). Data are presented as the mean ± SD (n = 3-4). *$p < 0.05$, **$p < 0.01$, ***$p < 0.001$, ****$p < 0.0001$ (compared with the R28^{Norm} group were performed using one-way analysis of variance followed by Tukeys post-hoc test).

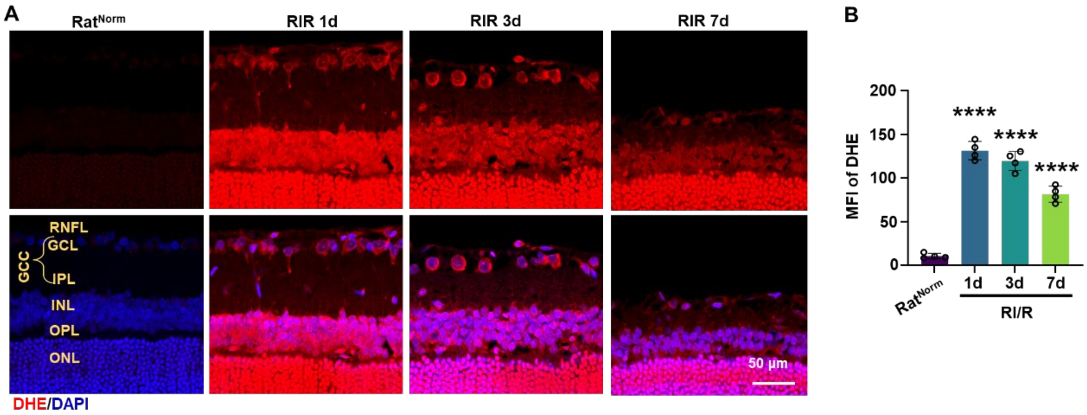

Figure S2. RI/R induces dynamic oxidative stress in rat retinas. A. Representative images of retinal sections stained with dihydroethidium (DHE, red) to detect superoxide production. The cell nucleus was stained by DAPI (blue). **B.** Quantification of DHE fluorescence intensity. Data are presented as the mean ± SD (n = 4 rats). ****$p < 0.0001$ (compared with the Rat^{Norm} group were performed using one-way analysis followed by Tukeys post-hoc test).

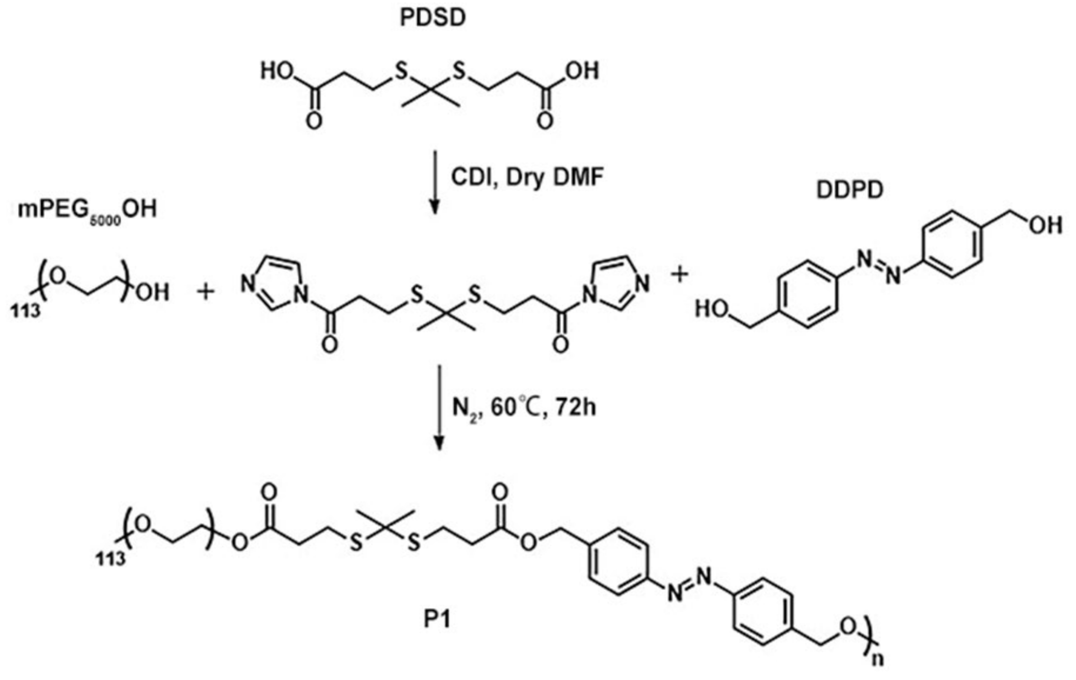

Figure S3. Schematic representation of the synthesis route for P1.

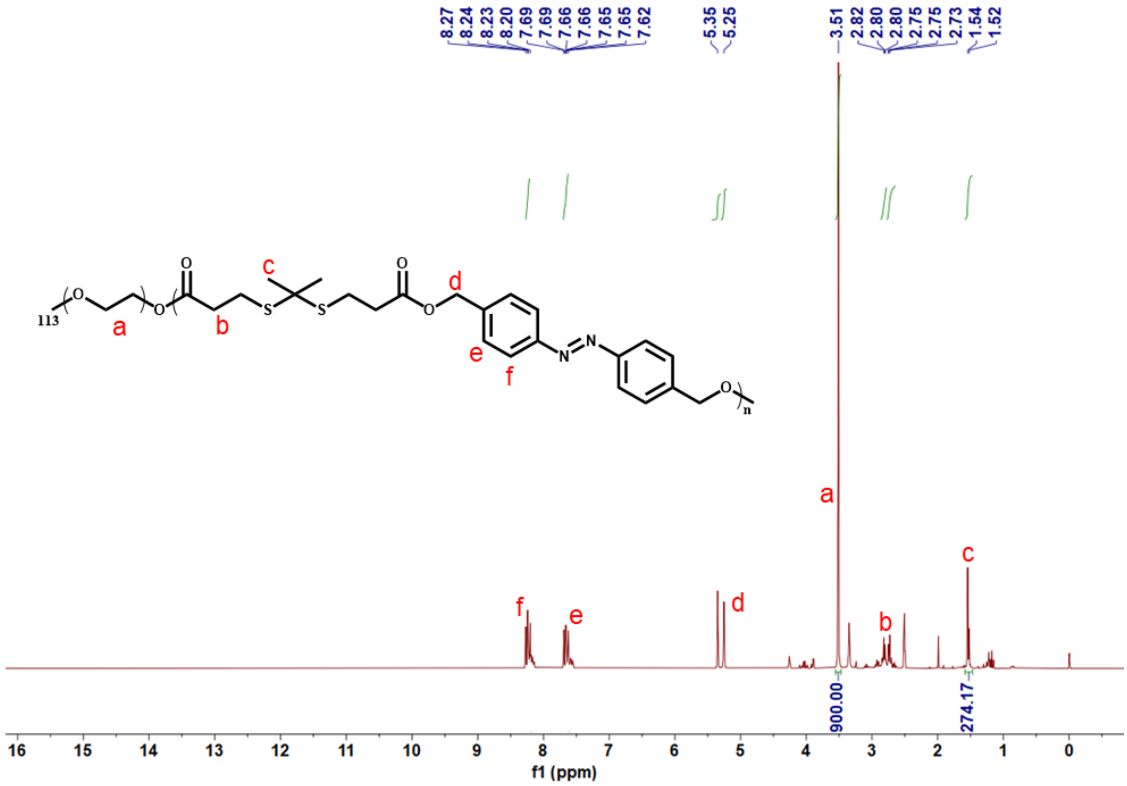

Figure S4. Characterization of P1 in DMSO-d6 by nuclear magnetic resonance (^{1}H-NMR) spectroscopy.

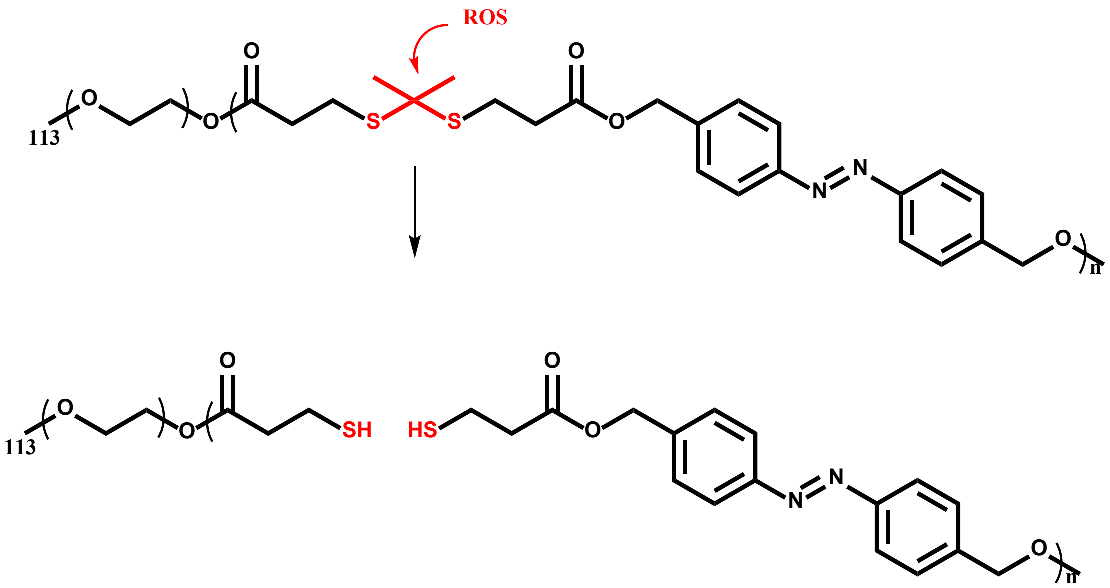

Figure S5. Mechanism of degradation of MT-NPs by accumulated ROS.

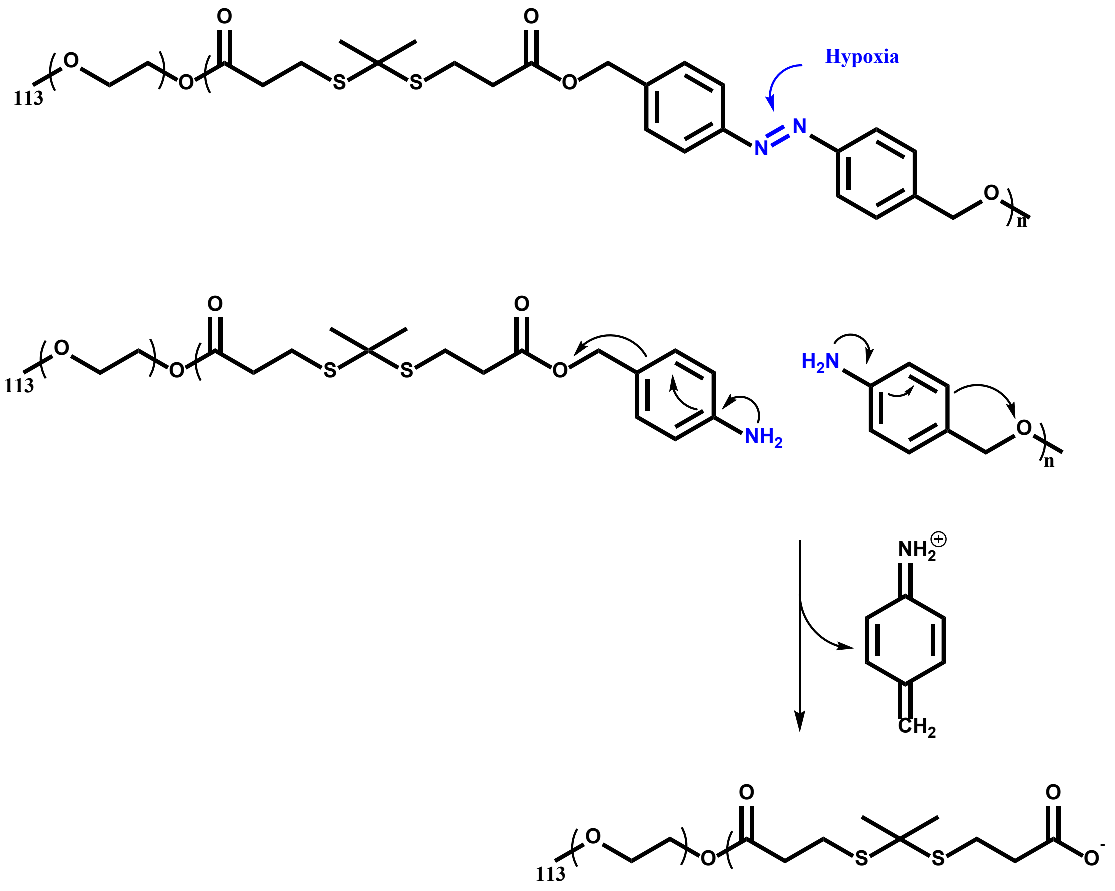

Figure S6. Mechanism of degradation of MT-NPs under hypoxia.

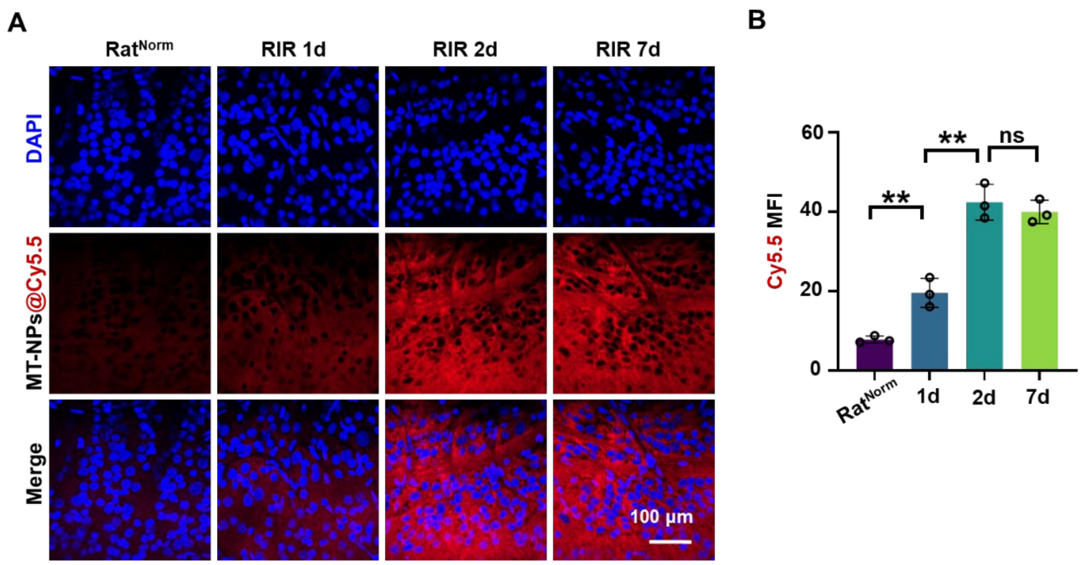

Figure S7. Intracellular uptake of MT-NPs@Cy5.5 by retinal cells. A. Representative images of rat retinal whole-mounts on day 1, 2, 7 after intravitreal injection of MT-NPs@Cy5.5. The red fluorescence came from Cy5.5 (red). The cell nucleus was stained by DAPI (blue). **B.** Quantification of mean fluorescence intensity (MFI) of MT-NPs@Cy5.5 in retinal whole-mounts. Data are presented as the mean ± SD (n = 3 rats). ns, not significant, $^{**}p < 0.01$ (comparisons between different groups were performed using one-way analysis of variance followed by Tukeys post-hoc test).

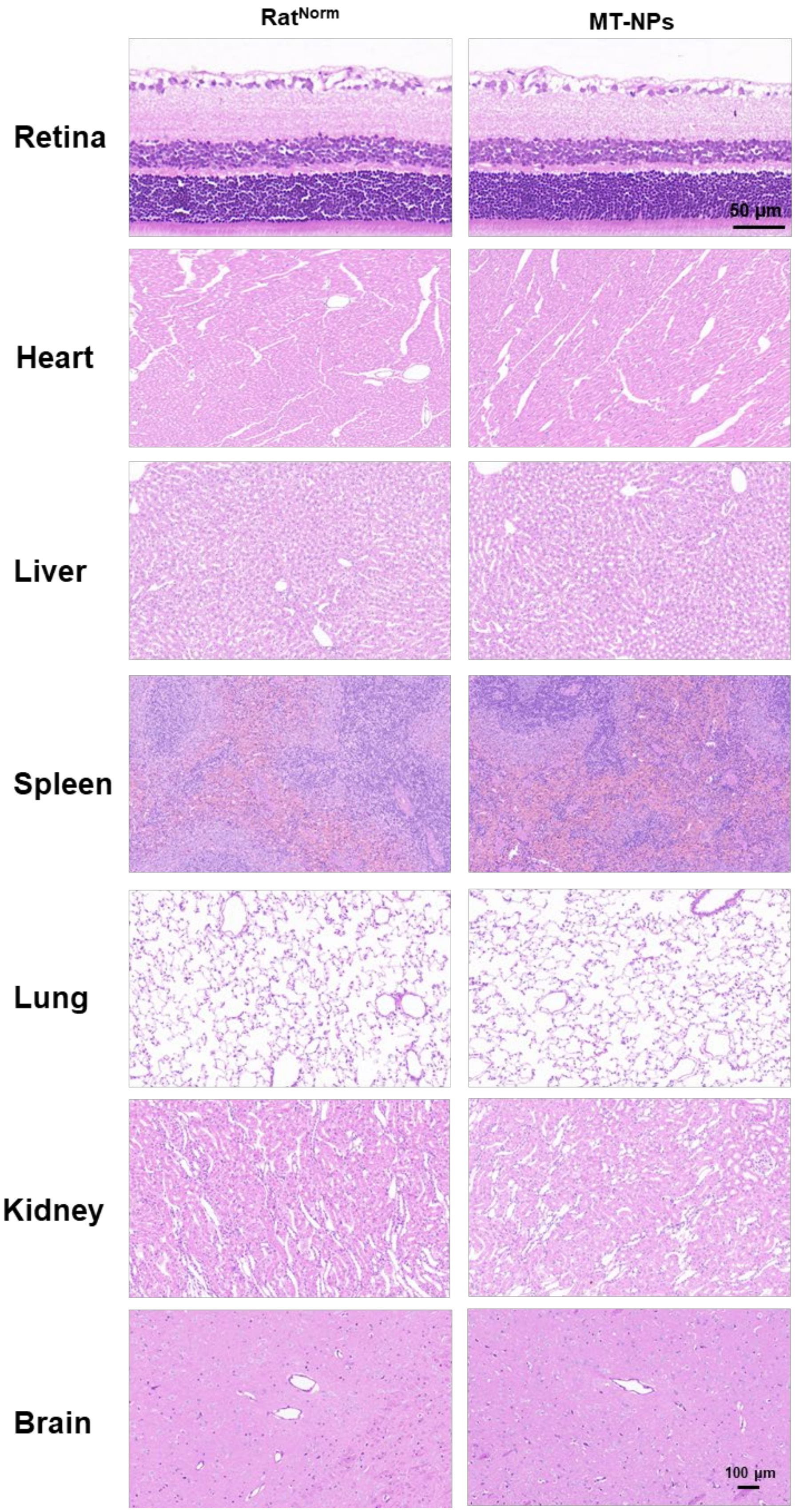

Figure S8. MT-NPs does not cause systemic vital organ damage on day 14 after intravitreal injection in rats (n = 3 rats).

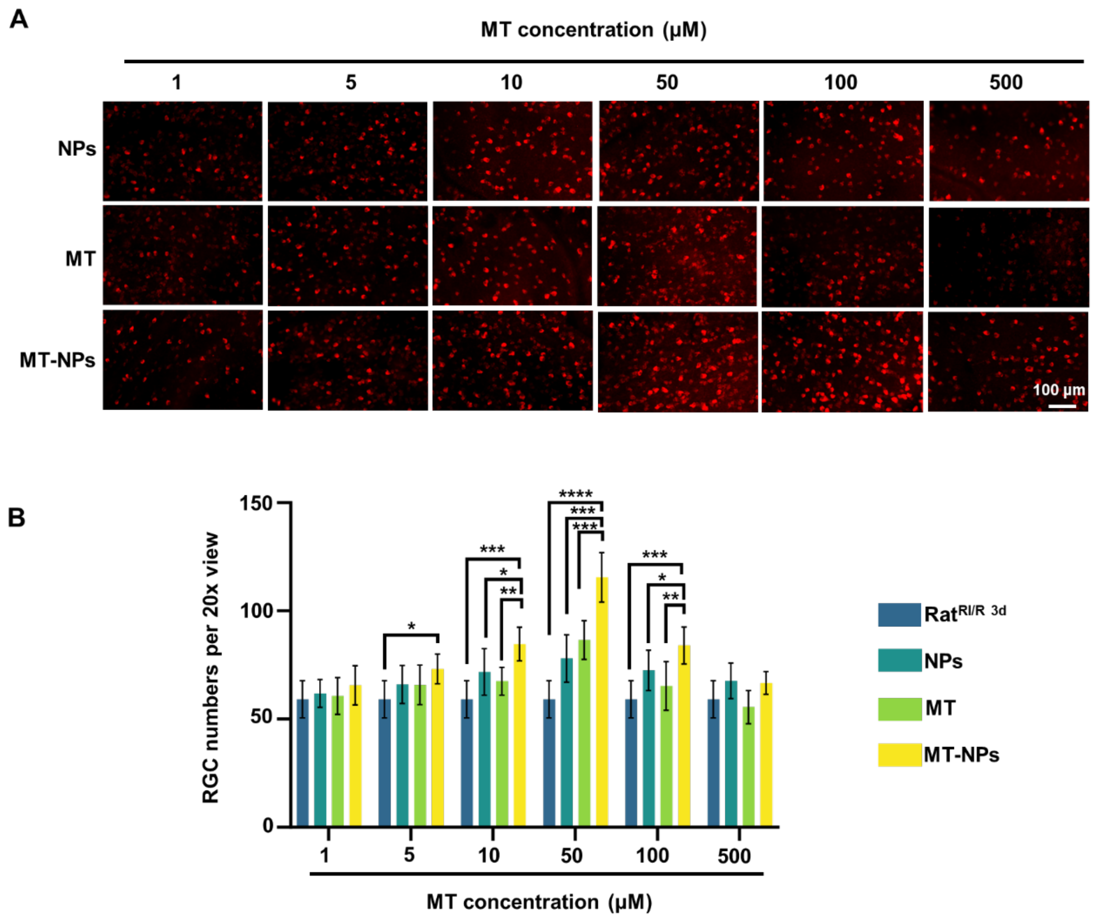

Figure S9. *In vivo* protection of rat$^{RI/R}$ with NPs, MT and MT-NPs at different concentrations. A. RBPMS labeled RGCs in flattening of rat retinas under different treatments. **B.** Quantification of RGCs per field of view. Data are presented as the mean ± SD (n = 4-6 rats). *$p < 0.05$, **$p < 0.01$, ***$p < 0.001$, ****$p < 0.0001$ (comparisons between different groups were performed using Student t-test).

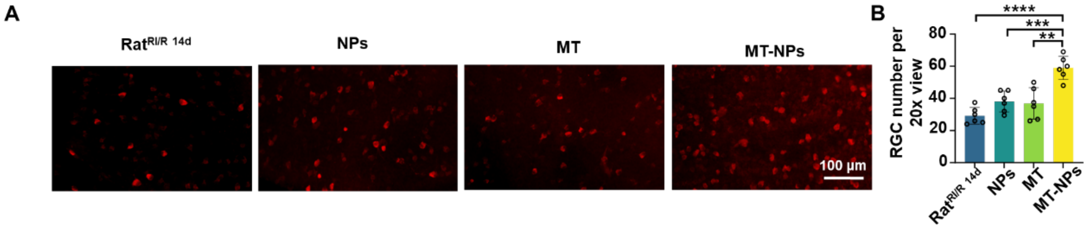

Figure S10. MT-NPs preserve RGCs on day 14 after RI/R injury. A. RBPMS labeled RGCs in flattening of rat retinas under different treatments. **B.** Quantification of RGCs per field of view. Data are presented as the mean ± SD (n = 6 rats). **$p < 0.01$, ***$p < 0.001$, ****$p < 0.0001$ (comparisons between different groups were performed using Student t-test).

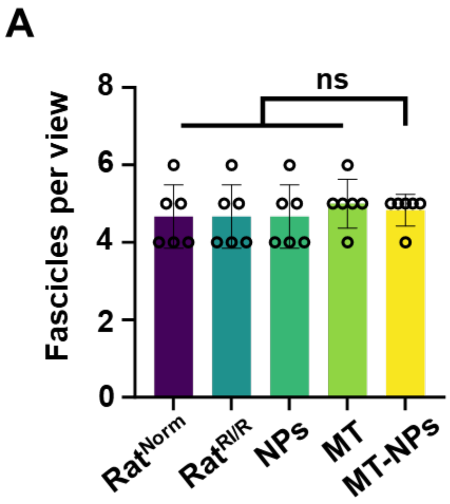

Figure S11. The number of fascicles per view in rat retinas. A. Data are presented as the mean ± SD (n = 6 rats). ns, not significant (comparisons between different groups were performed using Student t-test).

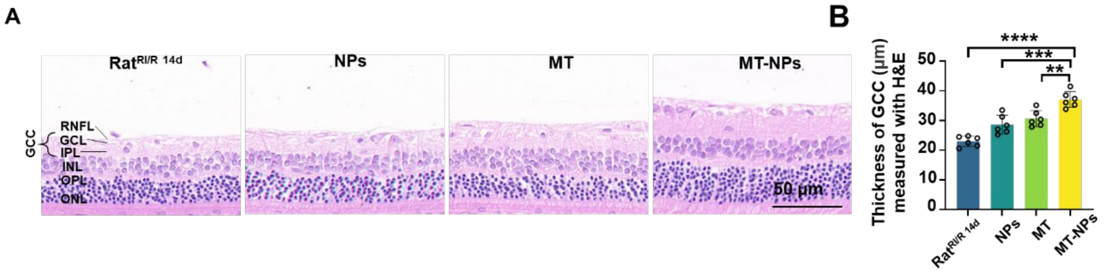

Figure S12. MT-NPs preserve retinal structure on day 14 following RI/R injury. A. H&E staining of rat retinas under different treatments. **B.** The thickness of GCC with different treatments measured via H&E staining (n = 6 rats). **p < 0.01, ***p < 0.001, ****p < 0.0001 (comparisons between different groups were performed using Student t-test).

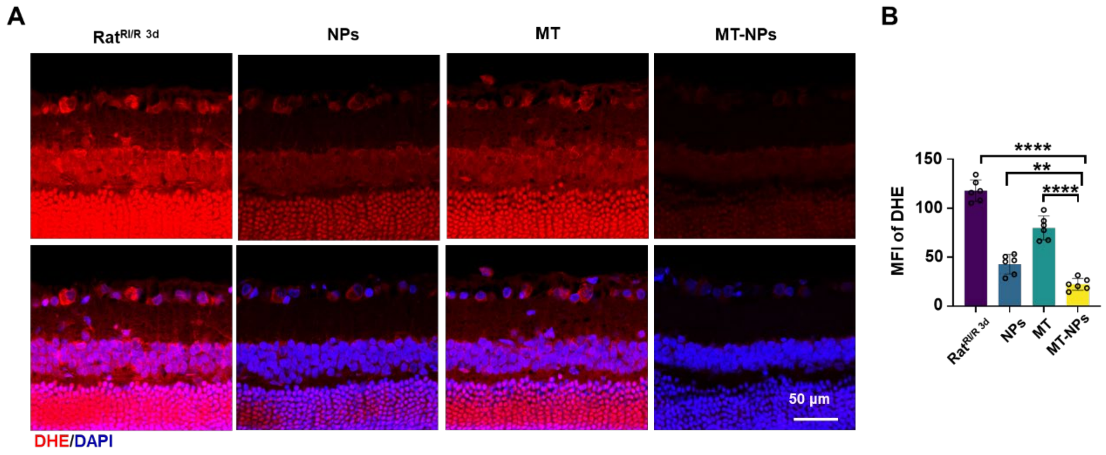

Figure S13. MT-NPs mitigate retinal oxidative stress *in vivo*. A. DHE staining (red) of retinal sections. Nuclei were stained with DAPI (blue). **B.** Quantification of DHE fluorescence intensity. Data are presented as the mean ± SD (n = 6 rats). $^{**}p < 0.01$, $^{****}p < 0.0001$ (comparisons between different groups were performed using Student t-test).

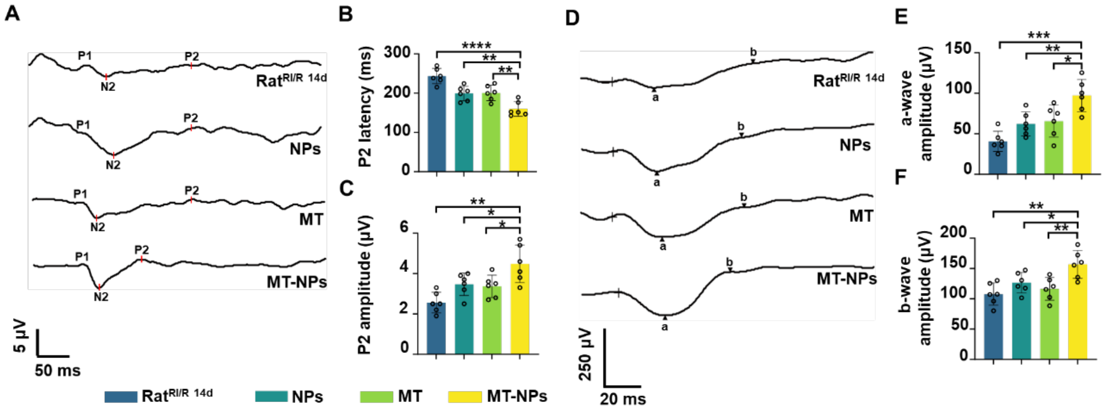

Figure S14. MT-NPs improve visual function on day 14 after RI/R injury. A. The FERG results of rats under various treatments. **B.** Quantification of FVEP P2 latency. **C.** Quantification of FVEP P2 amplitude. **D.** The FERG results of rats under various treatment. **E.** Quantification of FERG a-wave amplitudes. **F.** Quantification of FERG b-wave amplitudes. Data are presented as the mean ± SD (n = 6 rats). *$p < 0.05$, **$p < 0.01$, ***$p < 0.001$, ****$p < 0.0001$ (Comparisons between different groups were performed using Student t-test).

Table S1. Characterization data for P1.

	degree of polymerization	Drug loading content (%)	Encapsulation Efficiency (%)
P1	23	6.47	58
